# A solution structure analysis reveals a bent collagen triple helix in the complement activation recognition molecule mannan-binding lectin

**DOI:** 10.1016/j.jbc.2022.102799

**Published:** 2022-12-15

**Authors:** Hina Iqbal, Ka Wai Fung, Jayesh Gor, Anthony C. Bishop, George I. Makhatadze, Barbara Brodsky, Stephen J. Perkins

**Affiliations:** 1 Department of Structural and Molecular Biology, University College London, London, United Kingdom; 2 Center for Biotechnology and Interdisciplinary Studies, Rensselaer Polytechnic Institute, Troy, New York, USA; 3 Department of Biomedical Engineering, Science and Technology Center, Tufts University, Medford, Massachusetts, USA

**Keywords:** analytical ultracentrifugation, collagen, complement, atomistic modeling, molecular dynamics, small-angle neutron scattering, small-angle X-ray scattering, AUC, analytical ultracentrifugation, CD, circular dichroism, DSC, differential scanning calorimetry, MD, molecular dynamics, POG, the tripeptide sequence Pro-Hyp-Gly, *R*_*g*_, radius of gyration, SANS, small angle neutron scattering, SAXS, small angle X-ray scattering, MBL, mannan-binding lectin, T_m_, melting temperature

## Abstract

Collagen triple helices are critical in the function of mannan-binding lectin (MBL), an oligomeric recognition molecule in complement activation. The MBL collagen regions form complexes with the serine proteases MASP-1 and MASP-2 in order to activate complement, and mutations lead to common immunodeficiencies. To evaluate their structure-function properties, we studied the solution structures of four MBL-like collagen peptides. The thermal stability of the MBL collagen region was much reduced by the presence of a GQG interruption in the typical (X-Y-Gly)n repeat compared to controls. Experimental solution structural data were collected using analytical ultracentrifugation and small angle X-ray and neutron scattering. As controls, we included two standard Pro-Hyp-Gly collagen peptides (POG)_10-13_, as well as three more peptides with diverse (X-Y-Gly)n sequences that represented other collagen features. These data were quantitatively compared with atomistic linear collagen models derived from crystal structures and 12,000 conformations obtained from molecular dynamics simulations. All four MBL peptides were bent to varying degrees up to 85^o^ in the best-fit molecular dynamics models. The best-fit benchmark peptides (POG)_n_ were more linear but exhibited a degree of conformational flexibility. The remaining three peptides showed mostly linear solution structures. In conclusion, the collagen helix is not strictly linear, the degree of flexibility in the triple helix depends on its sequence, and the triple helix with the GQG interruption showed a pronounced bend. The bend in MBL GQG peptides resembles the bend in the collagen of complement C1q and may be key for lectin pathway activation.

The distinctive triple-helix motif is the defining feature of collagen proteins, which provide mechanical support and mediate cell signaling in the extracellular matrix. This collagen triple-helix motif also forms a domain in several host-defense proteins, including mannan-binding lectin (MBL), complement C1q and macrophage scavenger receptors. The standard collagen triple helix structure consists of three left-handed polyproline II helical chains twisted around each other to form a right-handed superhelix. This triple-helix structure is stabilized by the regular repeating sequence (Xaa-Yaa-Gly)_n_ which leads to the close packing of Gly residues from all three chains in the center of the superhelix (1, 2, 3). The Xaa and Yaa positions are exposed to solvent and are frequently occupied by the imino acids proline (Pro, P) and the posttranslationally modified hydroxyproline (Hyp, O), both of which promote triple helix stability (4, 5, 6). (Xaa-Yaa-Gly)_n_ sequences that are rich in Pro and Hyp are important for triple-helix stabilization and folding, while sequences with charged or hydrophobic residues play roles in recognition and biological activity (6, 7).

A perfect (Xaa-Yaa-Gly)_n_ repeating pattern is found in all fibrillar collagens, such as type I collagen in bone and tendon and type III collagen in skin. The replacement of a single Gly by a larger residue within a (Xaa-Yaa-Gly)n sequence has been found to be the cause of collagen-related diseases, including osteogenesis imperfecta, Ehlers Danlos syndrome IV, and Alports Disease (8). The replacement of a single Gly by any larger residue leads to reduced thermal stability and in some cases perturbed fibril formation or kinks (9). In contrast to fibrillar collagens, all nonfibrillar collagens, such as basement membrane type IV collagen, contain sites where the (Xaa-Yaa-Gly)_n_ repeating pattern is interrupted, a feature also found in triple-helix domains of host defense proteins (10, 11).

Triple-helix domains in host-defense proteins play key structural and interactive roles. MBL of the lectin pathway of complement activation binds to the sugar patterns on the surfaces of pathogenic microorganisms through its carbohydrate-recognition domains. Each MBL chain comprises a cysteine-rich region, a collagen region, an α-helical neck and a carbohydrate recognition domain (Fig. 1). The monomeric structural unit of a full-length native MBL molecule contains a homotrimeric triple helix, with a sequence pattern (Xaa-Yaa-Gly)_5_-Gln-Gly-(Xaa-Yaa-Gly)_10_. In this triple-helix domain, the (Xaa-Yaa-Gly)_n_ pattern is interrupted at one site by an incomplete triplet, QG, and similar breaks in repeating pattern have been shown to be highly destabilizing. Further, the triple-helices N terminal to the QG break self-associate to form MBL trimers to hexamers. MBL circulates in blood in complexes with the MBL-associated serine proteases (MASPs) (MASP-1, MASP-2, and MASP-3), and these proteases bind to MBL through its collagen region, close to the Gly-Gln-Gly (or GQG) interruption (Fig. 1) (12, 13). Gly substitutions by larger residues in the N-terminal part of the MBL triple-helix are a common cause of susceptibility to infections and are associated with defects in MBL oligomerization, decreased binding to its MASP ligands, and MBL deficiency in serum (14, 15, 16).

**Figure 1:**
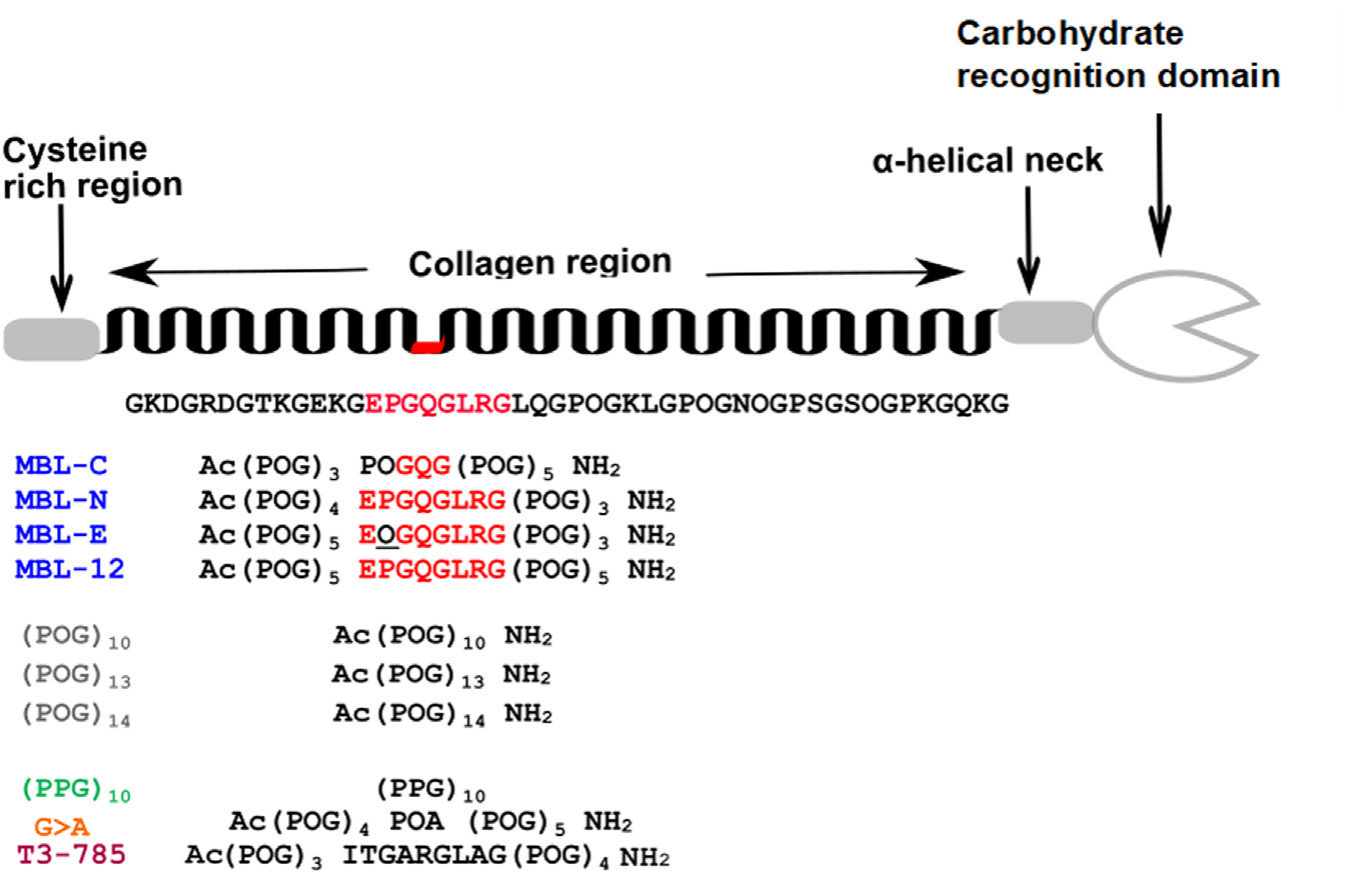
**Synthetic collagen peptides used in the study.** A cartoon of a single chain of mannan-binding lectin (MBL) monomer is shown at the top, with the collagen interruption region shown in *red*. Beneath, the blocked ends refer to N-terminal acetylation and C-terminal amidation. The peptides as shown correspond to those used in the AUC, SAXS, and SANS experiments below. From *top* to *bottom*, the sequences are shown for four peptides (*blue*) based on the MBL collagen region, namely MBL-C, MBL-N, MBL-E, and MBL-12. The corresponding sequence in native MBL is highlighted in *red*, flanked with (POG)_n_ triplets and blocked ends. The sequences of three of four standard peptides with blocked ends (POG)_10_, (POG)_13_, and (POG)_14_ are colored *gray*, whereas one standard unblocked peptide (PPG)_10_ is colored *green*. The peptide G > A containing a Gly>Ala substitution in (POG)_10_ with blocked ends is colored *orange*. The peptide T3-785 containing the partial sequence of near matrix metalloproteinase-1 collagenase cleavage site in type III collagen is colored *pink*. POG, the tripeptide sequence Pro-Hyp-Gly; SANS, small angle neutron scattering; SAXS, small angle X-ray scattering; AUC, analytical ultracentrifugation.

Synthetic peptides have been used to elucidate the molecular details of the collagen triple-helix through X-ray crystallography (17) and NMR (18) and to characterize physical chemical properties in solution (14). The sequence Pro-Hyp-Gly (POG) is the most common triplet and the most stabilizing sequence, and peptides including a significant number of repeating (POG)_n_ units will form a stable triple-helix. To better define the molecular flexibility of the collagen triple-helix, an atomistic modeling approach, in combination with analytical ultracentrifugation (AUC) and small-angle X-ray scattering (SAXS), was applied to a set of peptides consisting of repeating POG triplets of different lengths. The results indicated a small degree of inherent nonlinearity for the longer (POG)n peptides, despite the close packing of the three chains and the rigid imino acid side chains (19).

The application of complementary AUC, SAXS, and small-angle neutron scattering (SANS) experiments combined with molecular dynamics (MD) modeling (20) to triple-helical (POG)_n_ peptides (19) provides a starting point for investigating the effect of the QG interruption in the MBL triple-helix on molecular bending. A set of four peptides was designed to model the QG interruption within the triple-helix domain of MBL (Fig. 1 and Table 1), varying peptide length, the sequence immediately surrounding the QG interruption and hydroxylation of Pro. Breaks in the (Xaa-Yaa-Gly)n sequence, such as QG are known to destabilize the triple-helix. The effect of QG destabilizing feature on triple-helix flexibility was compared with the molecular flexibility of peptides containing three other features known to affect the stability of the triple-helix: prolyl hydroxylation, destabilizing Xaa and Yaa residues, and the replacement of one Gly by Ala (14, 21, 22). This combined experimental SAXS/AUC and modeling method was also applied here to three triple-helical peptides with destabilizing features whose high resolution crystal structures are published, namely (PPG)_10_, a peptide with no hydroxyproline (23); the G->A peptide in which a single Gly in (Pro-Hyp-Gly)_10_ is replaced by an Ala, to model Gly substitution mutations (24); and T3-785, a peptide carrying the imino acid poor human Type III collagen sequence near the unique MMP cleavage site (25) (Fig. 1 and Table 1). Defining the solution structural consequences in collagen model peptides which lack the standard (POG)_n_ sequence pattern as well as MBL peptides showed a relationship between collagen sequence and triple-helix linearity and can help explain the biological significance of sequence variations and breaks in MBL and collagens. Strikingly, one peptide containing the QG break flanked by two wildtype triplets showed a marked bending, while other destabilization features did not cause marked nonlinearity.

**Table 1:** The 10 synthetic collagen peptides of this study are listed

Collagen peptide	Mass (Da)	Full triplets	T_m_ (°C; CD)^a^	T_m_ (°C; DSC)	Sequence
MBL-C (MBL center)	7949	9	29	39	Ac-(POG)_4_-QG-(POG)_5_-NH_2_
MBL-N (MBL native)	8179	9	21	27	Ac-(POG)_4_-EPGQGLRG-(POG)_3_-NH_2_
MBL-E (MBL EOG)	9019	10	30	34	Ac-(POG)_5_-EOGQGLRG-(POG)_3_-NH_2_
MBL-12 (12 triplets)	10,574	12	n.a.	47	Ac-(POG)_5_-EPGQGLRG-(POG)_5_-NH_2_
(POG)_10_	8194	10	64, 60	70	Ac-(POG)_10_-NH_2_
(POG)_13_	10,652	13	n.a.	n.a.	Ac-(POG)_13_-NH_2_
(POG)_14_^b^	11,471	14	n.a.	n.a.	Ac-(POG)_14_-NH_2_
(PPG)_10_	8079	10	33	45	-(PPG)_10_-
G>A	8236	10	29	n.a.	Ac-(POG)_4_-POA-(POG)_5_-NH_2_
T3-785	8360	10	20	n.a.	Ac-(POG)_3_-ITGARGLAGPOG-(POG)_3_-NH_2_

Nine of these had their C and N termini blocked with acetyl and amide groups respectively. One peptide (PPG)_10_ with unblocked ends was studied.

n.a. not available; T_m_, the melting temperatures.

a The T_m_ values were derived from fits that assume a linear dependence of triple-helix ellipticity and a linear dependence of the unfolded monomer chain ellipticity, and then determining the temperature where Folding = 0.5.

b Only AUC data were acquired for this peptide.

## Results

### Design of the collagen peptides

To study conformational flexibility in the MBL collagen stalks, four MBL-related peptides were designed that included the GQG interruption flanked either by POG tripeptides or by the native human MBL triplets (Fig. 1). MBL-C (short for MBL-center) contained only the GQG interruption with repeating POG triplets on both sides, while MBL-N (short for MBL-native) included one human triplet sequence from MBL on each side of the GQG interruption (GEPGQGLRG). Pro residues in the Yaa positions of the triple-helix are typically posttranslationally hydroxylated to form Hyp, but amino acid sequencing showed that the Pro immediately N terminal to the MBL GQG interruption is not hydroxylated (26). To investigate the structural implications of this unusual Pro residue, a homologous peptide MBL-E (short for MBL-EOG) was synthesized in which the Pro is replaced by Hyp (GEOGQGLRG). To examine the effect of triple-helix length, a longer version of peptide MBL-N was designed, namely MBL-12, which included two additional POG triplets. Studies on the repeating POG peptides (POG)_6_, (POG)_8_, (POG)_10_, and (POG)_12_ were previously reported (19), and here studies on the collagen peptides (POG)_10_, (POG)_13_, and (POG)_14_ were again included as controls for the MBL peptide measurements.

To broaden our understanding of the relation between triple-helix thermal stability, triple-helix parameters, and molecular flexibility, the modeling/hydrodynamic studies below were also carried out on three peptides with distinct destabilizing features whose high-resolution crystal structures are available as benchmarks. An unblocked peptide (PPG)_10_ has Pro in all Yaa positions, rather than the more stabilizing Hyp (PPG)_10_ (23). The G > A peptide replaces a single Gly in (Pro-Hyp-Gly)_10_ by an Ala in order to model mutations, and such Gly replacements were shown to markedly decrease stability (24). Finally, peptide T3-785 carries the imino acid-poor human Type III collagen sequence near the unique matrix metalloproteinases cleavage region noted for its destabilizing Xaa and Yaa residues (25) (Fig. 1 and Table 1).

### Thermal stability of the MBL collagen peptides

**Figure 2:**
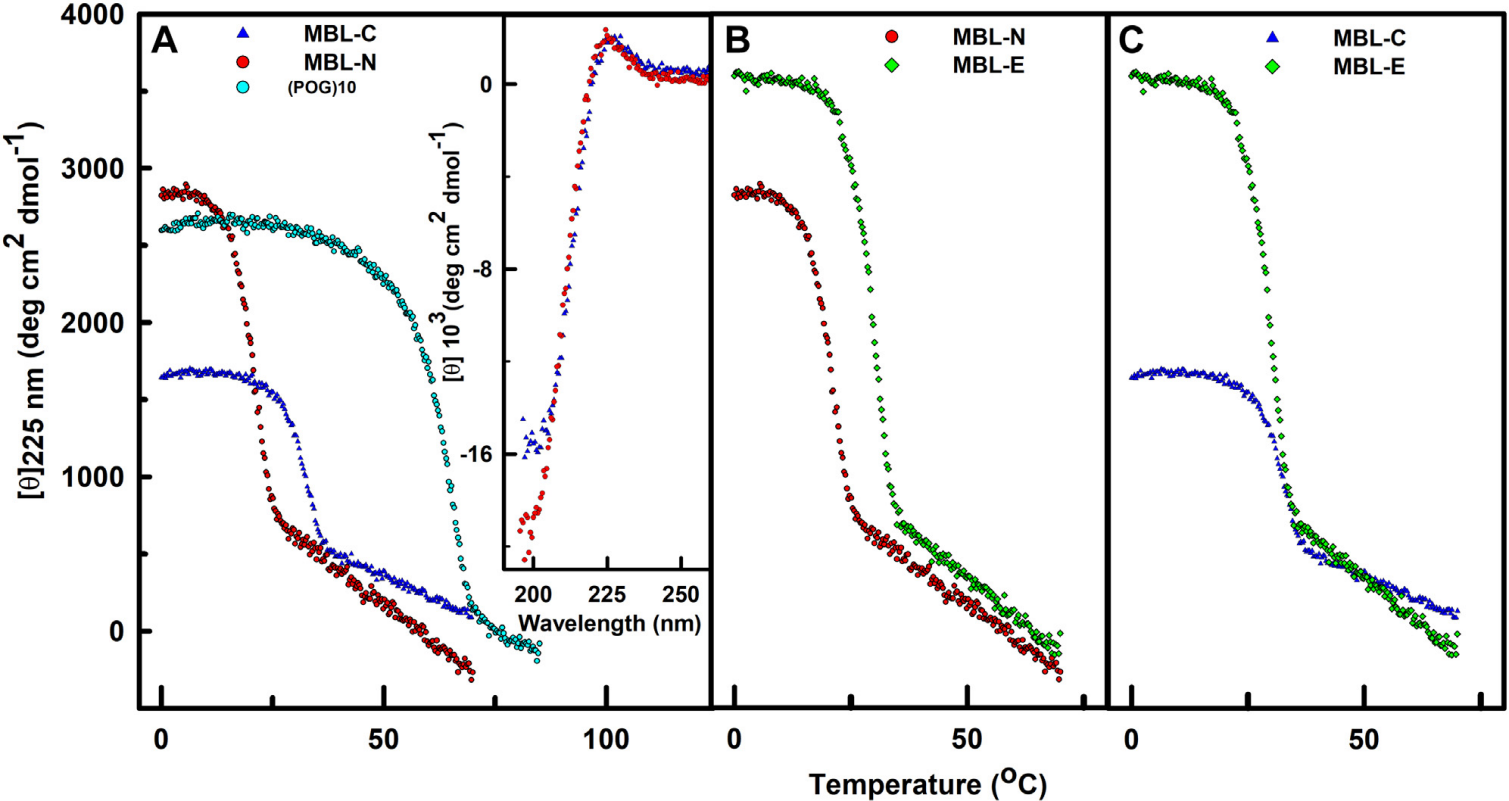
**Circular dichroism study of the melting temperatures of the MBL collagen peptides.** The wavelength at 226 nm is monitored in all samples between approximately 5 to 70 °C. The insets in panel *A* show the full CD spectrum between 195 and 255 nm at 20 °C. *A*, comparison of MBL-C (*blue*), MBL-N (*red*), and (POG)_10_ (*cyan*) as in Figure 1. *B*, comparison of MBL-N (*red*) and MBL-E (*green*) except that MBL-E has one fewer N-terminal POG triplet compared to Figure 1. *C*, comparison of MBL-C (*blue*) and MBL-E (*green*) except that MBL-E has one fewer POG triplet at the N terminus compared to Figure 1. POG, the tripeptide sequence Pro-Hyp-Gly; MBL, mannan-binding lectin; CD, circular dichroism.

Circular dichroism (CD) spectroscopy was carried out on the four MBL collagen peptides, as well as on a (POG)_10_ control, to explore the effect of the GQG interruption and how the identity of residues in the collagen triplet Xaa and Yaa positions influenced triple-helix stability (Fig. 1). Thus temperature-dependent CD studies were performed at a fixed wavelength of 226 nm on the four MBL collagen peptides, as well as on (POG)_10_. All the MBL peptides showed typical triple-helix CD features, with a maximum at 226 nm (inset, Fig. 2*A*), and a minimum at 197 to 198 nm, and the ratio of the ellipticity of the positive peak to the negative peak was close to that expected for a fully triple-helical peptide (27). The intensities at a wavelength of 226 nm were used because it represents the maximum characteristic of the triple-helix and undergoes the largest change during denaturation, from a positive signal to a negative signal. The minimum at 198 nm which is seen for the native triple-helix is also seen for the denatured single chain structures, with a decreased magnitude, and was not used to follow the transitions. The thermal transitions of the triple-helix structures were accordingly obtained by monitoring the ellipticity at 226 nm (Fig. 2, *A*–*C*).

The melting curves were obtained under identical conditions, at a very slow heating rate, as previously described (28). Extensive studies indicate that the thermal transitions at this slow rate approximate equilibrium, but do not fully reach it, and are kinetically limited (26, 29). It was not possible to slow the heating rate down enough to reach equilibrium because this led to aggregation. Thus, the melting temperatures T_m_ obtained under the standard conditions in our lab reflect well with the comparative thermal stability of different triple-helical peptides. Both the MBL-N and MBL-C peptides formed triple helices and have melting temperatures of 21 °C and 32 °C, respectively (Table 1). The MBL-C peptide was thus more stable than MBL-N as there was a 9 °C difference in the melting temperature (Fig. 2*A*). In contrast, the control peptide (POG)_10_ showed a higher melting temperature of 64 °C, which compares well with an earlier value of 60 °C (14) and showed that the GQG interruption in MBL destabilized the collagen helix (Fig. 2*A*). To check the effect of the Pro or Hyp residue next to the GQG interruption, the MBL-N peptide was compared with a slightly modified MBL-E peptide in order to compare the same number of POG repeats in both peptides (Fig. 2*B*). The replacement of Pro with Hyp increased the melting temperature from 21 °C to 30 °C, showing a stabilization caused by Hyp in MBL-E. Finally, MBL-C and MBL-E were compared to see whether the positioning of the POG triplets before or after the GQG interruption conferred stability (Fig. 2*C*). There, both peptides showed almost the same melting temperature of 29 °C and 32 °C respectively, thus the positioning of the POG triplets had little effect on stability. For completion, the corresponding T_m_ values of 33 °C, 29 °C, and 20 °C from our earlier studies of (PPG)_10_, G > A and T3-785, respectively, were included in Table 1 (14, 25, 28). These additional values support the premise that modifications of the collagen helix reduce its melting temperatures and destabilize it.

**Figure 3:**
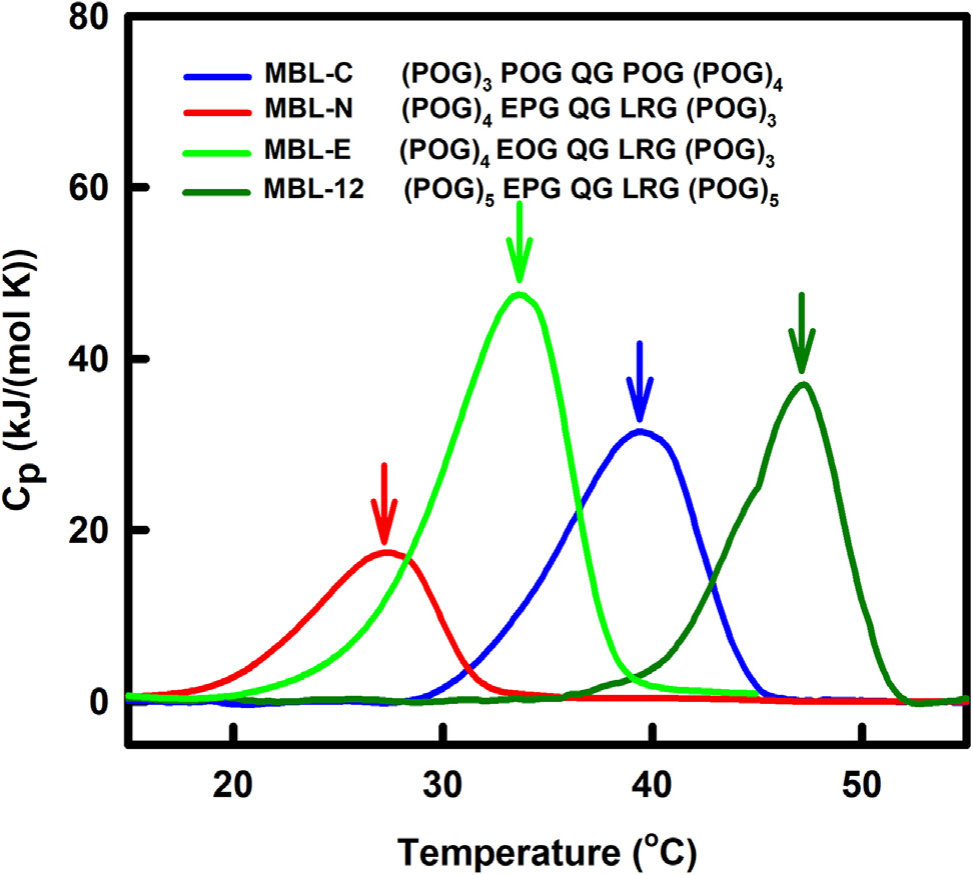
**Differential scanning calorimetry study of the melting temperatures of the MBL collagen peptides**. The four peptides correspond to MBL-C, MBL-N, and MBL-E with one fewer N-terminal POG compared to Figure 1, and MBL-12. Asymmetric features can arise from nonequilibrium conditions in part of the transition. The melting temperatures are *arrowed*. MBL, mannan-binding lectin; POG, the tripeptide sequence Pro-Hyp-Gly.

Differential scanning calorimetry (DSC) was performed on the MBL peptides to confirm the outcome of the CD studies (Fig. 3). Single peaks were seen for the MBL peptides as desired, while the observed asymmetric DSC profiles, can arise under nonequilibrium conditions, rather than representing multiple transitions (29). The MBL-N, MBL-E, MBL-C, and MBL-12 peptides gave melting temperatures of 27 °C, 34 °C, 39 °C, and 47 °C (Table 1). That for (POG)_10_ was previously measured as 70 °C (26, 30), while that for (PPG)_10_ was 45 °C (29). The thermal transition values obtained by DSC followed the same trend as the CD values, with slightly higher T_m_ values than those measured by CD, these differences being attributed to the faster DSC heating rate under nonequilibrium conditions (Table 1). There was good concordance in the measured stabilities of the four MBL peptides, and it was concluded that the GQG interruption does in fact destabilize the MBL collagen helix. Additional destabilization in the MBL collagen region was conferred by the replacement of the expected Hyp residue in the POG triplet with Pro just before the GQG interruption in the native MBL sequence (MBL-E). Additional POG triplets stabilized the MBL peptides. The formation of stable triple-helical structures by all four MBL peptides laid the ground for the hydrodynamic and scattering characterization of these molecules below.

### Analytical ultracentrifugation of the collagen peptides

First, experimental data on the 10 collagen peptides in solution was acquired by a combination of AUC, SAXS, and SANS. Second, 12,000 physically realistic atomistic models for each of these 10 collagen triple helices were produced using MD. A comparison through filtering of these theoretical models against the experimental SAXS and SANS data identified the best-fit structures for each collagen triple helix in solution. This procedure followed our procedures for atomistic scattering modeling (19, 20).

**Figure 4:**
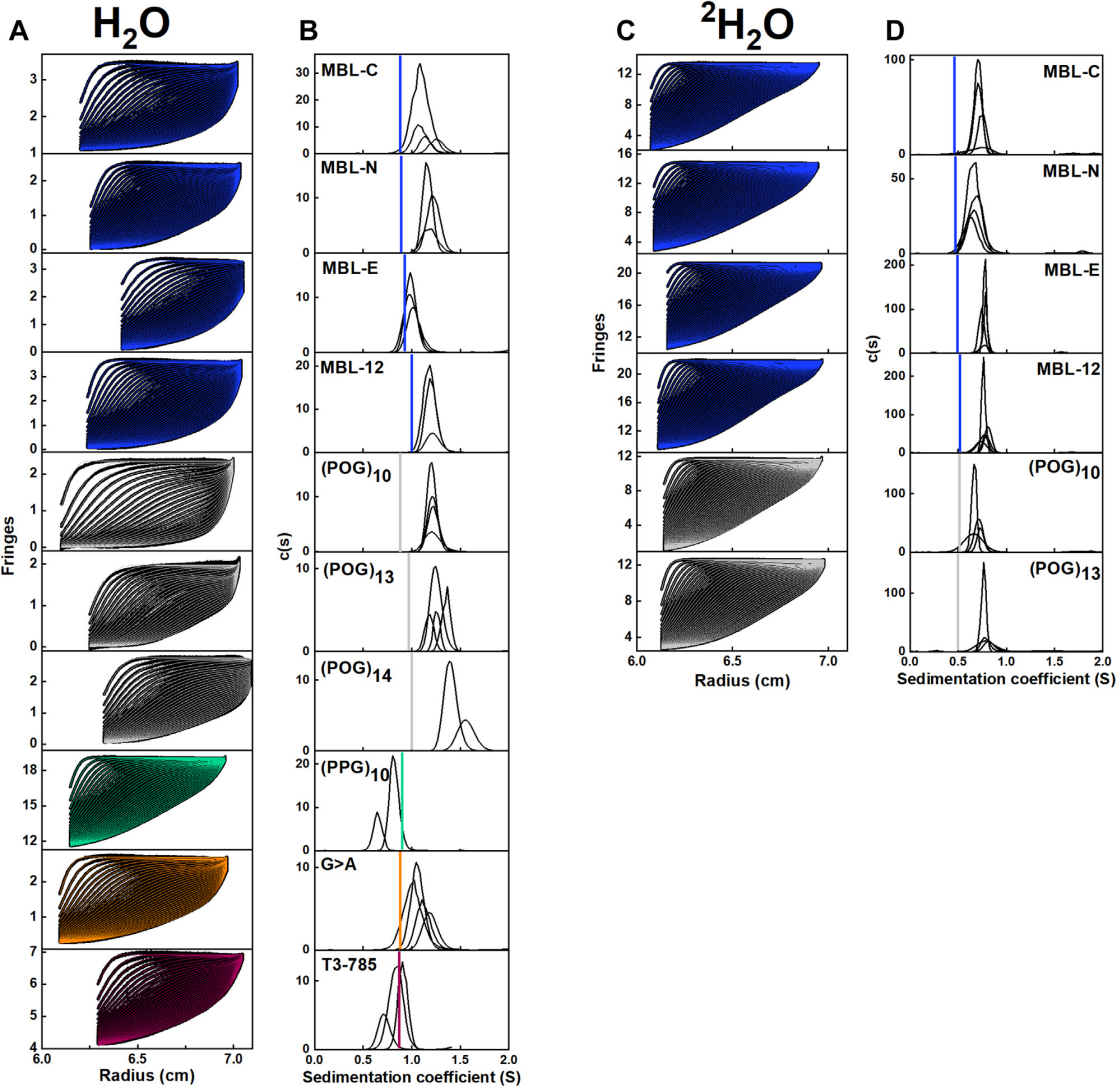
**Sedimentation velocity analyses of the 10 collagen peptides**. *A* and *C*, the experimental sedimentation boundaries of the collagen peptides obtained in 137 mM PBS or 20 mM L-His buffer in H_2_O and in 20 mM L-His buffer with ^2^H_2_O, respectively. The experimental boundary scans (*black lines*) were acquired at 20 °C and 50,000 rpm using interference/absorbance optics at 222 nm. Up to 45 scans (every third scan) were fitted using SEDFIT (colored lines to follow Fig. 1). The meniscus positions varied depending on sample availability. *B* and *D*, the corresponding peaks are shown from the size distribution analyses *c(s)* in H_2_O and ^2^H_2_O, respectively. The vertical colored lines correspond to the theoretical *s*_*20,w*_ values calculated from the linear crystal-derived models for the helices (Table 2). The 2 to 4 *c(s)* curves from the concentration series for each sample are shown together in each panel to indicate the reproducibility of the velocity analyses. The peptide concentrations for experiments in H_2_O were 0.25 to 1 mg/ml for MBL-C, 0.25 to 1 mg/ml for MBL-N, 0.25 to 1.25 mg/ml for MBL-E, 0.25 to 1.25 mg/ml for MBL-12, 0.25 to 1 mg/ml for (POG)_10_, 0.20 to 0.75 mg/ml for (POG)_13_, 0.40 to 0.80 mg/ml for (POG)_14_, 1 to 2.5 mg/ml for (PPG)_10_, 0.40 to 0.95 mg/ml for G > A, and 0.3 to 1.1 mg/ml for T3-785, respectively. The concentrations for experiments in ^2^H_2_O were 0.94 to 3.7 mg/ml for MBL-C, 1.5 to 4 mg/ml for MBL-N, 0.75 to 4 mg/ml for MBL-E, 1 to 4 mg/ml for MBL-12, 1 to 4 mg/ml for (POG)_10_, and 1 to 3.5 mg/ml for (POG)_13_ respectively. MBL, mannan-binding lectin; POG, the tripeptide sequence Pro-Hyp-Gly.

The rate of sedimentation of a macromolecule under high centrifugal force is dependent on its shape and size. A globular molecule sediments faster compared to an elongated molecule (31). Sedimentation velocity experiments were conducted on the 10 collagen peptides in light water buffer, and six of these peptides in heavy water (Fig. 4, *A* and *C*). All 10 peptides formed stable triple helices in both phosphate and histidine buffers at 20 °C. The size distribution *c(s)* analyses resulted in good fits to the sedimentation boundaries and produced single narrow distinct peaks for each collagen helix demonstrating structurally monodispersity (Fig. 4, *B* and *D*). The root-mean-square residuals from SEDFIT were satisfactory at 0.01 to 0.02 for the interference data fits and up to 0.04 for the absorbance data fits. In all 10 cases, the absence of peaks at the lowest *S* values confirmed that there was no dissociation of the helices into single peptides. The experimental sedimentation coefficient *s*_*20,w*_ values were calculated from the peak positions in the *c(s)* analyses. For the MBL peptides, their averaged *s*_*20,w*_ values were 1.20 *S*, 1.15 *S*, 1.09 *S* and 1.30 *S* for MBL-C, MBL-N, MBL-E, and MBL-12, respectively, in 137 mM PBS buffer (Table 2). MBL-E sedimented slightly more slowly than the smallest MBL-C and MBL-N peptides, but overall, the rate of sedimentation increased with size. For the standard collagen peptides, the averaged *s*_*20,w*_ values were 1.28 *S*, 1.30 *S*, 1.61 *S*, and 0.95 *S* for (POG)_10_, (POG)_13_, (POG)_14_, and (PPG)_10_, respectively, in 137 mM PBS (Table 2). For the (POG)_n_ peptides, the averaged *s*_*20,w*_ values increased with the number of (POG) repeats, which reflected the combination of both the increasing mass and lengths of the helices. The proline-rich peptide (PPG)_10_ sedimented slower (∼0.33 *S* less) than the equivalent length collagen peptide (POG)_10_. The average rates of sedimentation for the G > A and T3-785 peptide were 1.19 *S* and 0.95 *S*, respectively. However, (PPG)_10_ sedimented almost at the same rate as the Type III T3-785 peptide of the same length, suggesting similar hydrodynamic properties for these two peptides (Fig. 4 and Table 2). With the use of heavy water as a control measurement (and for use in the neutron experiments below), the *s*_*20,w*_ values increased to 1.42 *S*, 1.31 *S*, 1.51 *S*, 1.55 *S*, 1.39 *S*, and 1.59 *S* for MBL-C, MBL-N, MBL-E, MBL-12, (POG)_10_, and (POG)_13_, respectively (Fig. 4 and Table 2). The sedimentation rates were higher in heavy water buffer due to the different density of the hydration shell surrounding the peptides in heavy water (32).

**Table 2:** Summary of the sedimentation experiments and modeling for 10 collagen peptides

Collagen peptides	AUC (light water)	AUC (heavy water)	Theoretical *s*_*20, w*_ (S)	
	*s*_*20, w*_ (S)	*s*_*20, w*_ (S)	Linear model(hydrated)	Best-fit MD model
MBL-C	1.20 ± 0.01	1.42 ± 0.01	0.88	0.96
MBL-N	1.15 ± 0.06	1.31 ± 0.03	0.89	0.97
MBL-E	1.09 ± 0.04	1.51 ± 0.10	0.93	1.02
MBL-12	1.30 ± 0.03	1.55 ± 0.02	1.00	1.10
(POG)_10_	1.28 ± 0.10	1.39 ± 0.10	0.88	0.96
(POG)_13_	1.30 ± 0.04	1.59 ± 0.09	0.97	1.11
(POG)_14_	1.61 ± 0.10	n.a.	1.00	n.a.
(PPG)_10_	0.95 ± 0.03	n.a.	0.90	0.97
G>A	1.19 ± 0.10	n.a.	0.88	0.96
T3-785	0.95 ± 0.08	n.a.	0.87	0.96

The theoretical *s*_*20, w*_ values were predicted using HYDROPRO. The frictional ratios from the SEDFIT analyses ranged between 1.2 for the shorter collagen peptides up to 1.5 for the longer ones.

n.a. Not available.

**Figure 5:**
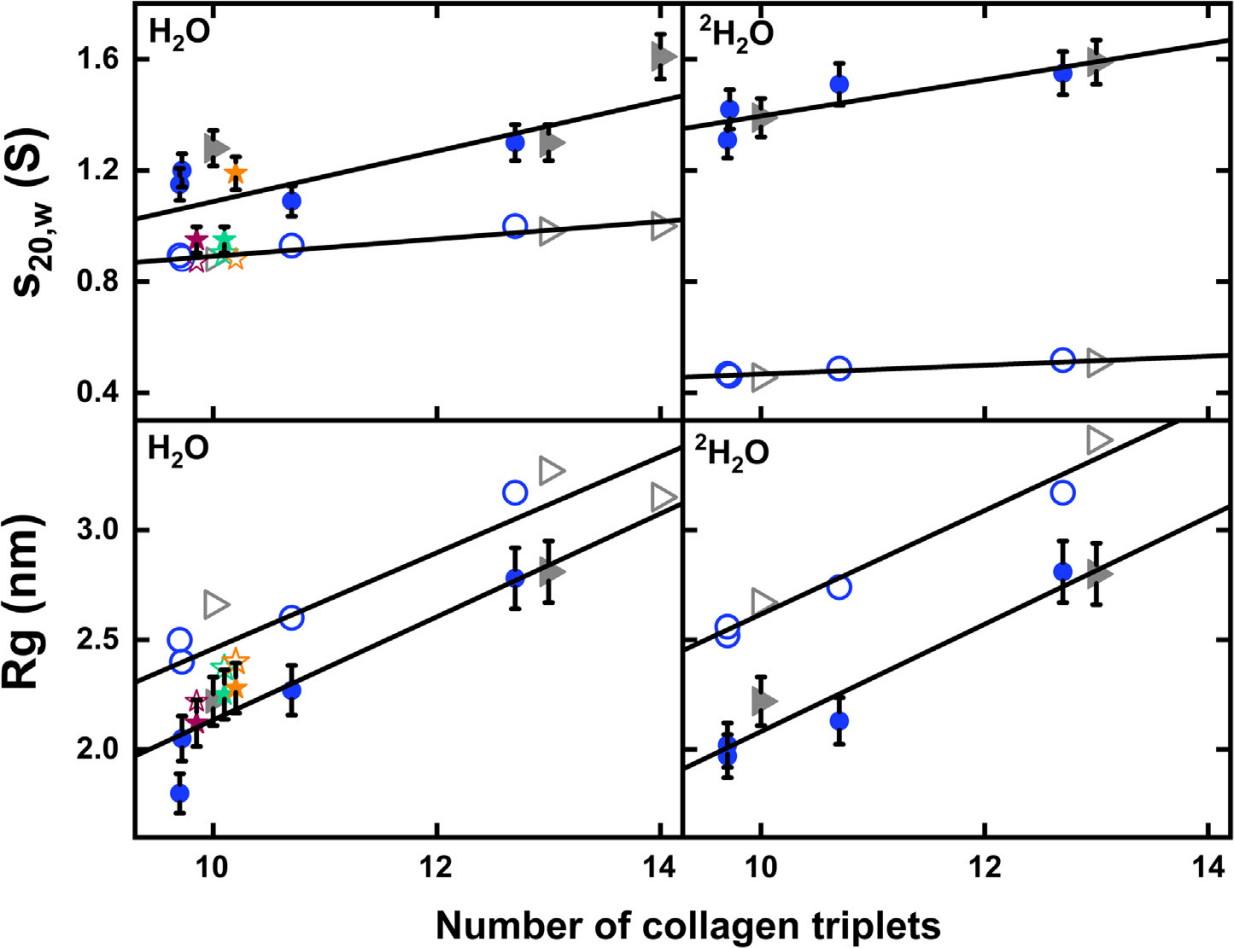
**Comparison of the experimental and crystallographic *s***_***20,w***_**and *Rg* values**. The *left* and *right panels* display data for the collagen helices in 137 mM PBS or 20 mM L-histidine buffer in H_2_O and 20 mM L-histidine buffer in ^2^H_2_O, respectively. The *top panels* compare the *s*_*20,w*_ values against the peptide length. The bottom panels compare the *Rg* values against the peptide length. The noninteger values correspond to partial triplets (Fig. 1). The theoretical values were calculated from the nine linear crystal-derived models (*open symbols*). The experimental *s*_*20,w*_ and *Rg* values (*filled symbols with error bars*; Table 2) were subjected to linear fits (*black lines*). To follow Figure 1, *blue circles* represent the MBL-C, MBL-N, MBL-E, and MBL-12 peptides with 9.67, 9.67, 10.67, and 12.67 triplets, respectively, *gray triangles* represent the (POG)_10_, (POG)_13_, and (POG)_14_ peptides, the *green star* represents the unblocked (PPG)_10_ peptide, the *orange star* represents the blocked G > A peptide, and the *pink star* represents the unblocked T3-785 peptide. MBL, mannan-binding lectin; POG, the tripeptide sequence Pro-Hyp-Gly.

Theoretical sedimentation coefficients *s*^*0*^_*20,w*_ were calculated from the crystal-derived linear structures with hydrated and unhydrated parameters, using HYDROPRO (version 10.0) (33). The AUC experimental data in light water were compared with these calculated *s*^*0*^_*20,w*_ values to assess their divergence. The consistently greater experimental *s*_*20,w*_ values for the collagen peptides (filled data points) compared to the theoretical values for linear structures (unfilled data points) is consistent with some bent nonlinear collagen structures existing in solution (Fig. 5 and Table 2).

### X-ray and neutron solution scattering of the collagen peptides

**Figure 6:**
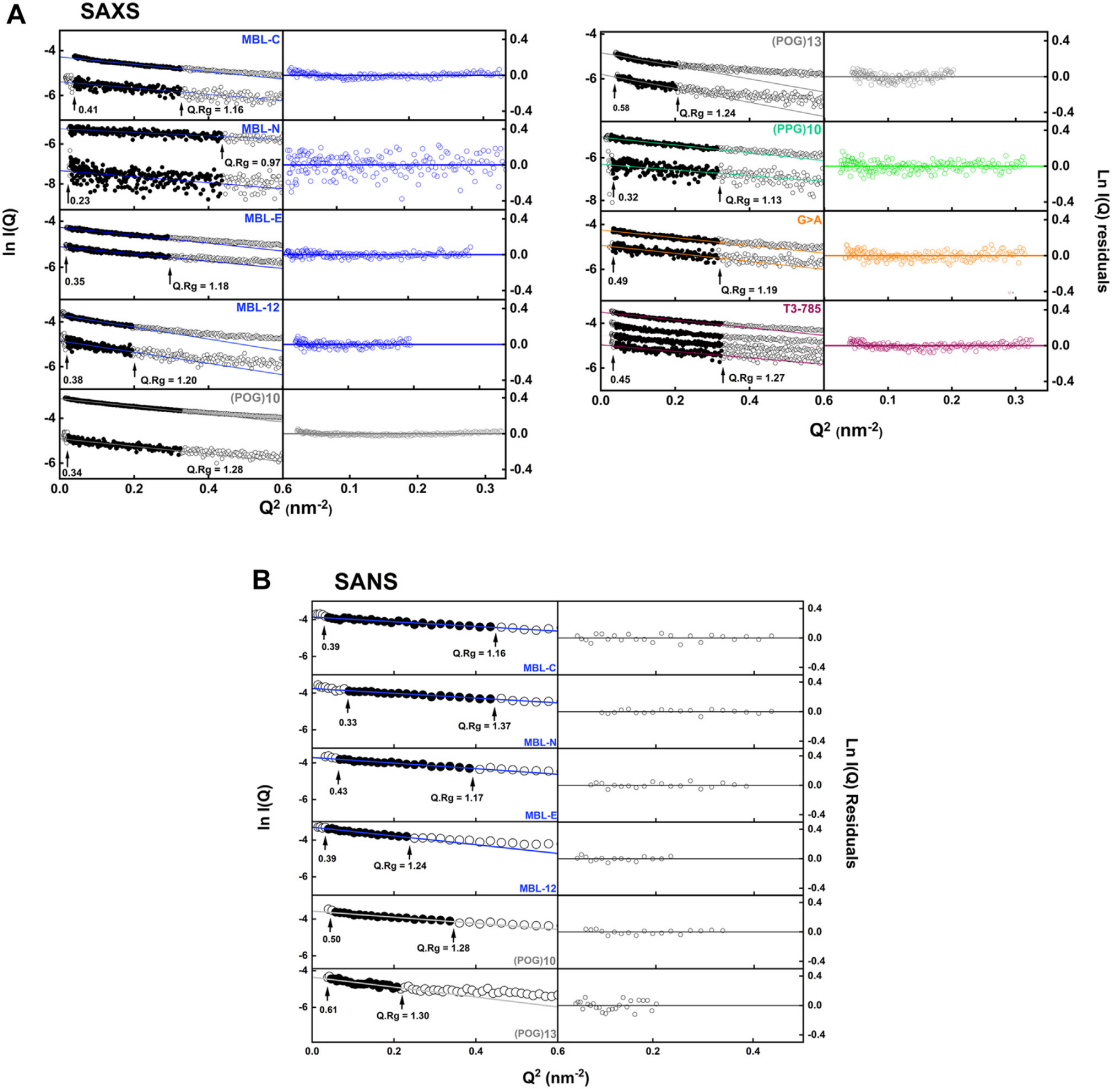
**Experimental X-ray and neutron Guinier*R***_***g***_**for the collagen peptides**. The peptide concentrations ranged between 1 and 2.5 mg/ml for SAXS and 4.0 and 5.5 mg/ml for SANS. *A*, the SAXS Guinier *R*_*g*_ plots at low *Q* values are shown in two columns. To the *left*, the filled symbols (*black*) denote the *Q* ranges used to determine the Guinier *R*_*g*_ from the linear fits (*colored lines*) of the *I*(*Q*) curves (open symbols). The *Q.R*_*g*_ limits for each fit are indicated with *arrows*. The *Q* ranges of the Guinier SAXS fits were between 0.20 and 0.57 nm^−1^ for MBL-C, 0.16 to 0.66 nm^−1^ for MBL-N, 0.16 to 0.54 nm^−1^ for MBL-E and 0.14 to 0.44 nm^−1^ for MBL-12. For the (POG)_n_ peptides, the *Q* ranges of the Guinier SAXS fits were successively reduced from 0.15 to 0.57 nm^−1^ for (POG)_10_ to 0.21 to 0.45 nm^−1^ for (POG)_13_. The SAXS *Q* ranges were between 0.16 and 0.56 nm^−1^ for (PPG)_10_, 0.18 and 0.56 nm^−1^ for G > A, and 0.20 and 0.57 nm^−1^ for T3-785. To the right, the residuals of the Guinier fits are shown relative to the fitted line. *B*, for the SANS Guinier *R*_*g*_ plots, the *Q* ranges of the corresponding fits were 0.20 to 0.57 nm^−1^ for MBL-C, 0.20 to 0.66 nm^-1^ for MBL-N, 0.24 to 0.58 nm^−1^ for MBL-E, 0.14 to 0.44 nm^−1^ for MBL-12 0.24 to 0.58 nm^-1^ for (POG)_10_ and 0.21 to 0.45 nm^-1^ for (POG)_13_. MBL, mannan-binding lectin; POG, the tripeptide sequence Pro-Hyp-Gly; SANS, small angle neutron scattering; SAXS, small angle X-ray scattering.

Solution scattering experiments characterized the average structure of the collagen peptides in terms of their overall length and width in solution. Synchrotron SAXS datasets were obtained for the 10 collagen peptides (Fig. 1) in two buffers (137 mM PBS, pH 7.4, or 20 mM L-histidine, pH 6.0). Our initial datasets were measured in PBS buffer and were prone to small nonspecific sample aggregation; the change to the histidine buffer removed this effect. The Guinier analyses of the SAXS curves gave linear plots from which the radii of gyration *R*_*g*_, a measure of macromolecular elongation, were derived (Fig. 6).

For the SAXS analyses, the *Q* ranges of the Guinier fits were similar, starting between 0.14 and 0.20 and ending at 0.44 to 0.66 nm^−1^ for the four MBL peptides. (Fig. 6*A*). For (POG)_14,_ the initial Guinier plots indicated high aggregation from the increased intensities at low Q values, and this was excluded from further analysis. For the two (POG)_n_ peptides, the *Q* ranges of the Guinier fits were successively reduced from 0.15 to 0.57 nm^−1^ for (POG)_10_ to 0.21 to 0.45 nm^−1^ for (POG)_13_. For the remaining three peptides, the *Q* ranges of the Guinier fits were all similar between 0.16 and 0.56 nm^−1^ for (PPG)_10_, 0.18 and 0.56 nm^−1^ for G > A, and 0.20 and 0.57 nm^−1^ for T3-785. Overall, the Guinier regions became reduced with increased length of the collagen peptides. The SAXS experimental *R*_*g*_ values calculated from the Guinier analyses ranged between 1.8 nm and 2.8 nm (Table 3) and showed low standard deviations. The *R*_*g*_ values were seen to increase with increasing length (Fig. 5), this being consistent with the AUC results in light water, which also demonstrated increased *s*_*20,w*_ values with collagen size. Comparison of the R_g_ values for (POG)_10_ here with that reported previously (19) showed a decrease to 2.22 ± 0.10 nm (Table 3) from 2.29 ± 0.02 nm previously, which was the same within error and demonstrated consistency.

**Table 3:** Summary of the SAXS and SANS experiments for nine collagen peptides

Collagen peptides	X-rays				Neutrons			
	*Guinier R*_*g*_ (nm)	*Real space R*_*g*_ (nm)	*M* (nm)	*L* (nm)	*Guinier R*_*g*_ (nm)	*Real space R*_*g*_ (nm)	*M* (nm)	*L* (nm)
MBL-C	2.05 ± 0.10	2.42 ± 0.01	1.18	8.7	1.97	2.38	0.95	8.7
MBL-N	1.65 ± 0.03	n.a.	1.18	7.1	2.02	2.31	0.95	9.0
MBL-E	2.27 ± 0.10	2.35 ± 0.01	1.18	10	2.13	2.54	0.95	9.7
MBL-12	2.80 ± 0.05	2.98 ± 0.02	1.31	12.5	2.81	3.04	1.0	12.5
(POG)_10_	2.23 ± 0.03	2.45 ± 0.00	1.27	8.5	2.22	2.59	1.0	10.5
(POG)_13_	2.80 ± 0.15	3.15 ± 0.23	1.23	9.5	2.85	3.09	0.95	10.0
(PPG)_10_	2.25 ± 0.10	2.75 ± 0.02	1.19	9	n.a.	n.a.	n.a.	n.a.
G>A	2.28 ± 0.02	2.64 ± 0.03	1.15	11	n.a.	n.a.	n.a.	n.a.
T3-785	2.22 ± 0.10	2.46 ± 0.14	1.28	10	n.a.	n.a.	n.a.	n.a.

n.a. Not available.

SANS datasets in heavy water buffers were obtained for six collagen peptides, namely MBL-C, MBL-N, MBL-E, MBL-12, (POG)_10_, and (POG)_13_. The Guinier plots for (POG)_14_ indicated excessive aggregation again at low *Q* values and were also excluded from further analysis. For neutrons, the *Q* ranges of the individual Guinier fits were similar (Fig. 6*B*). The SANS experimental *R*_*g*_ values were 1.97 nm for MBL-C, 2.02 nm for MBL-N, 2.13 nm for MBL-E, 2.81 nm for MBL-12, 2.22 nm for (POG)_10_, and 2.80 nm for (POG)_13_, again with low standard deviations (Table 3). The trend of an increase in *R*_*g*_ values with peptide length (Fig. 5) supported the above AUC and SAXS results. The SANS Guinier R_g_ values were similar to the SAXS R_g_ values.

**Figure 7:**
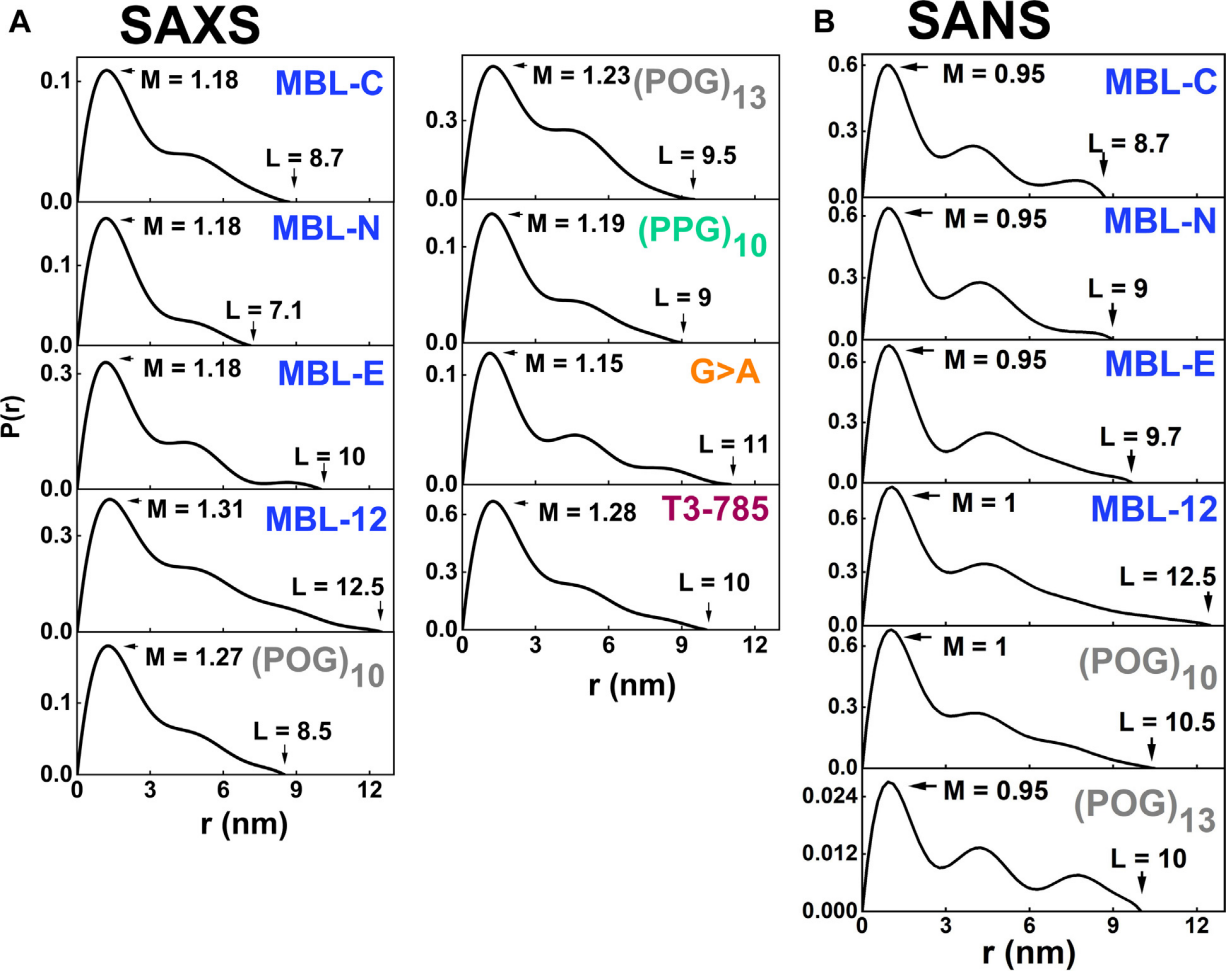
**Experimental X-ray and neutron pair distance distribution *P(r)* analyses for the collagen peptides**. *A* and *B*, the distance distribution curves for the collagen peptides from the experimental SAXS and SANS data are shown in two panels. The *r* values at the maximum lengths *L* (nm) and peak maxima *M* (nm) are indicated by *arrows*. The vertical scales are multiplied by 1000 for reason of clarity. MBL, mannan-binding lectin; POG, the tripeptide sequence Pro-Hyp-Gly; SANS, small angle neutron scattering; SAXS, small angle X-ray scattering.

Pair-distance distribution analyses *P(r)* of the full-length scattering *I(Q)* curves provided real space determinations of the *R*_*g*_ values (Table 3 and Fig. 7). The appearance of the *P(r)* curves was affected by noise in the *I(Q)* datasets that was attributed to the comparatively low molecular masses in use for the scattering experiments (Table 1). Nonetheless, good *P(r)* curves were obtained with *R*_*g*_ values that were comparable with the Guinier *R*_*g*_ values. The *P(r)* analyses provided the maximum length *L* of each collagen helix which corresponded to the value of *r* when the *P(r)* curve reaches zero. From the SAXS analyses, the maximum length *L* values were 8.7 nm for MBL-C, 7.1 nm for MBL-N, 10 nm for MBL-E, 12.5 nm for MBL-12, 8.5 nm for (POG)_10_, 9.5 nm for (POG)_13_, 9 nm for (PPG)_10_, 11 nm for G > A, and 10 nm for T3-785, respectively (Table 3). The *P(r)* peak maximum provided values for *M*, the most common interatomic distance in each collagen molecule, and its value reflects the width of the collagen triple helix. Here, the *M* values were 0.86 nm for MBL-C, 0.81 nm for MBL-N, 0.81 nm for MBL-E, 0.94 nm for MBL-12, 0.88 nm for (POG)_10_, 0.90 nm for (POG)_13_, 1.28 nm for (PPG)_10_, 0.86 nm for G > A, and 0.90 nm for T3-785 respectively. For MBL-12, the relatively small *L* and larger *M* values suggested some bending with increase in length. The increases in the *L* values were consistent with the increased length of the collagen peptides.

From the corresponding SANS *P(r)* analyses, the *L* values were 7.5 nm for MBL-C, 7.6 nm for MBL-N, 9.7 nm for MBL-E, 12.5 nm for MBL-12, 10.5 nm for (POG)_10_, and 10.0 nm for (POG)_13_ (Table 3). The *P(r)* curves gave *R*_*g*_ values that were comparable with the Guinier *R*_*g*_ values, although the comparison was against limited by noise in the *I(Q)* curves. The trends in the SANS *L* values followed that of the SAXS *L* values. Minor perturbations resulted in the form of an abrupt end to the *P(r)* curve at large *r* values or small extra *P(r)* peaks (Fig. 7*B*). The *M* values were 0.47 nm for MBL-C, 0.45 nm for MBL-N, 0.49 nm for MBL-E, 0.53 nm for MBL-12, and 0.57 nm for (POG)_10._ The SANS *M* values were noticeably reduced in heavy water compared to the SAXS data, and this was attributed to the near invisibility of the hydration shell in SANS experiments with heavy water (34).

### Comparison of the experimental SAXS and SANS data with the linear collagen models

Overall structural information on the peptides was obtained through the *R*_*g*_ calculation from the scattering curve *I(Q)* at low *Q* values. The experimental SAXS and SANS *R*_*g*_ values were compared with the theoretical *R*_*g*_ values calculated from the crystal-derived linear models (Experimental procedures) for the collagen peptides (Fig. 5). The theoretical *R*_*g*_ values were calculated for each of the linear crystal-derived curves using the same *Q* range used for the experimental Guinier curve fits for reason of consistency (Fig. 6). Given that SAXS visualizes hydrated protein structures, standard hydration shells were added to the linear structures. The resulting theoretical *R*_*g*_ values were 2.19 nm for MBL-C, 2.22 nm for MBL-N, 2.37 nm for MBL-E, 2.88 nm for MBL-12, 2.39 nm for (POG)_10_, 2.90 nm for (POG)_13_, 2.32 nm for (PPG)_10_, 2.42 nm for G > A, and 2.27 nm for T3-785 (Table 4). Comparison with the experimental SAXS *R*_*g*_ values (Table 3) showed that the theoretical *R*_*g*_ values were larger in all cases than the experimental *R*_*g*_ values (Fig. 5). This difference indicated that the linear models were too long to account for the experimental data, in turn indicating that the collagen peptides showed bending in their solution structures. The same comparison was made for the SANS *R*_*g*_ values, where this time SANS observes unhydrated structures because the hydration shell is almost invisible (34). Comparison of the experimental *R*_*g*_ values with the theoretical ones again showed that the theoretical ones were again larger, indicating bending of the linear collagen structures in solution (Fig. 5).

**Table 4:** Summary of the molecular modeling of nine collagen peptides from SAXS and SANS

Collagen peptide	*R*_*g*_ (nm)				*M* (nm)			*L* (nm)			R factors (%)			
	Linear model		Best-fit MD model		Linear model	Best-fit MD model		Linear model	Best-fit MD model		Linear model		Best-fit MD model	
	SAXS	SANS	SAXS	SANS		SAXS	SANS		SAXS	SANS	SAXS	SANS	SAXS	SANS
MBL-C	2.19	2.24	2.31	2.19	0.98	1.12	1.10	9.0	8.9	7.8	5.0	11.9	2.8	11.5
MBL-N	2.22	2.21	1.73	2.18	0.97	1.50	0.98	8.6	6.8	8.8	16.9	10.4	14.3	9.8
MBL-E	2.37	2.43	2.43	2.30	0.99	1.40	1.40	10.0	9.2	8.1	12.0	12.4	6.5	12.4
MBL-12	2.88	2.80	2.96	2.69	1.07	1.50	1.50	11.3	11.1	10.1	7.4	13.6	6.8	11.7
(POG)_10_	2.39	2.33	2.29	2.36	1.00	1.20	1.20	9.3	8.9	8.3	4.2	12.3	3.0	7.4
(POG)_13_	2.90	3.26	3.05	2.99	1.00	1.29	1.17	11.6	11.6	10.8	7.9	13.4	7.3	11.7
(PPG)_10_	2.32	n.a.	2.39	n.a.	1.00	1.28	n.a.	8.8	9.0	n.a.	5.7	n.a.	6.1	n.a.
G>A	2.42	n.a.	2.48	n.a.	0.96	1.28	n.a.	9.3	9.0	n.a.	3.3	n.a.	3.0	n.a.
T3-785	2.27	n.a.	2.29	n.a.	0.95	1.40	n.a.	8.7	9.0	n.a.	9.1	n.a.	5.9	n.a.

Analyses of the crystal-derived linear models and the best-fit MD-simulated models of nine collagen peptides (all except (POG)_14_). The *R*_*g*_, and the *M* and *L* values were calculated from the models using SCT *R*_*g*_, and GNOM *P(r)* analyses, respectively. The goodness-of-fit *R* factors were calculated by comparing the experimental scattering curves with those calculated for the linear and best-fit MD structures.

n.a. Not available.

**Figure 8:**
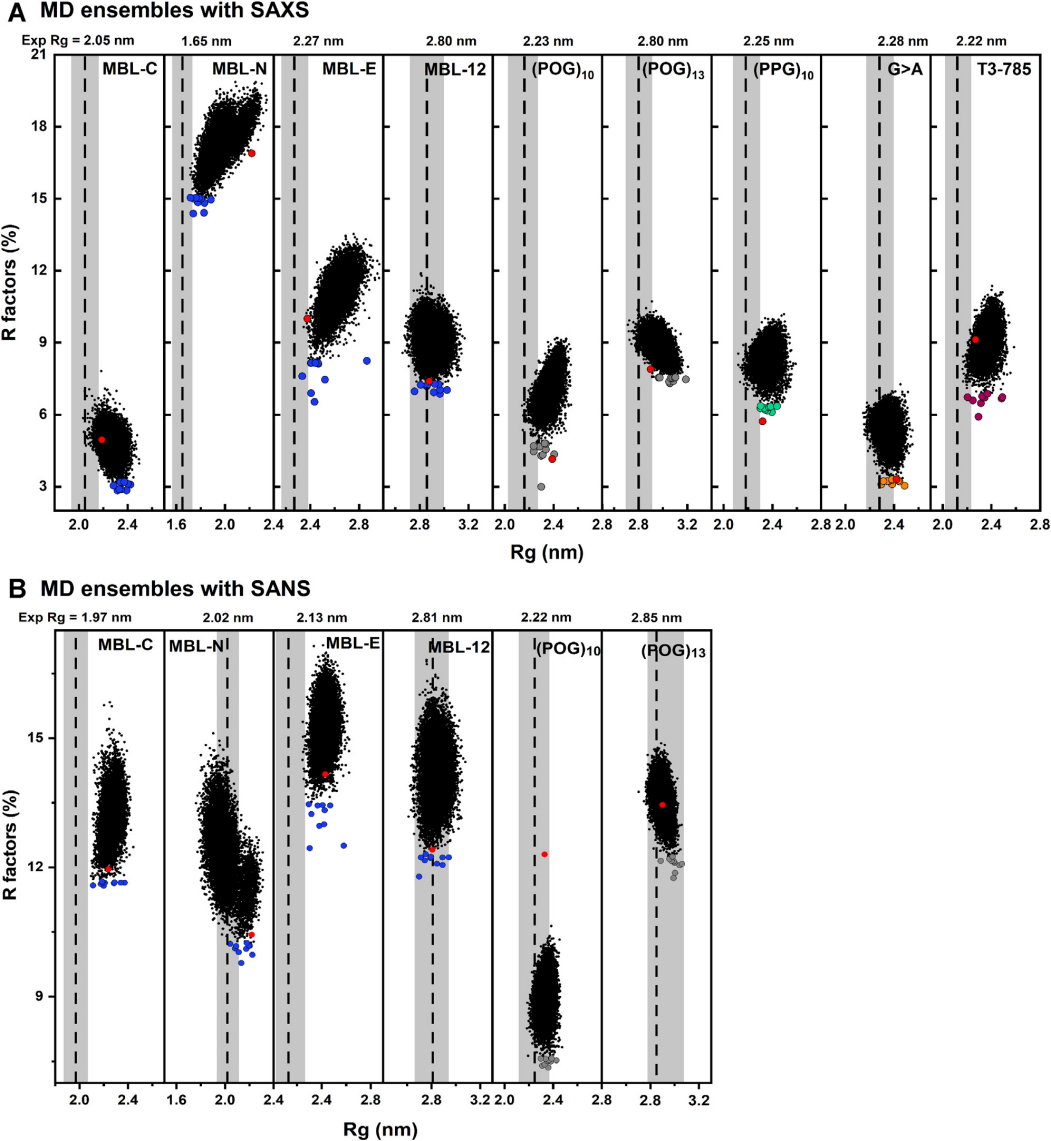
**Comparison of the molecular dynamics (MD) ensembles with the experimental scattering data**. *A*, 12,000 *R* factors were compared with X-ray *R*_*g*_ values computed for nine collagen models (MBL-C, MBL-N, MBL-E, MBL-12, (POG)_10_, (POG)_13_, (PPG)_10_, G > A, and T3-785). *B*, *R* factors of models compared with neutron *R*_*g*_ values calculated for six collagen models (MBL-C, MBL-N, MBL-E, MBL-12, (POG)_10_, and (POG)_13_). All models in an ensemble are shown as *black dots*. The 10 best-fit structures with the lowest *R* factors are shown as *filled symbols*, *blue* for MBL-C, MBL-N, MBL-E, and MBL-12; *gray* for (POG)_10_ and (POG)_13_; *green* for (PPG)_10_; *orange* for G > A, and *pink* for T3-785 collagens. The *R*_*g*_ and *R* factor values for each of the crystal-derived linear models are shown in *red circles*. The *dashed lines* represent the experimentally calculated *R*_*g*_ values, and the *shaded bands* represent ± 5% error range in these values. MBL, mannan-binding lectin; POG, the tripeptide sequence Pro-Hyp-Gly.

The scattering curve at large *Q* values offer further structural information on the macromolecule in solution. The full experimental and theoretical SAXS *I(Q)* curves were superimposed upon each other. The goodness-of-fit *R* factor monitors the degree of deviation between the two scattering curves. Visually the superimposition showed good agreements (Fig. 8, *A* and *B*). However, on comparing the SAXS curves against those for the linear structures, the *R* factors were comparatively larger than expected. They were 5.0% for MBL-C, 14.3% for MBL-N, 12.0% for MBL-E, 7.4% for MBL-12, 4.2% for (POG)_10_, 7.9% for (POG)_13_, 5.7% for (PPG)_10_, 3.3% for G > A, and 9.1% for T3-785 (Table 4). Exclusion of the hydration parameters for the linear crystal-derived models in order to compare these with the SANS experiment also resulted in high *R* factors of 11.9% for MBL-C, 10.4% for MBL-N, 12.4% for MBL-E, 13.6% for MBL-12, 12.3% for (POG)_10_, and 13.4% for (POG)_13_. Given that the crystal-derived structures were very close to linear, the higher *R* factors and poorer curve fits with increasing peptide length suggested nonlinearity in the helices (Table 4). This divergence indicated that the collagen peptides become bent in solution and less linear.

### MD simulations and curve fits for the collagen peptides

In order to model the scattering curves with greater precision, MD simulations to determine physically realistic atomistic structures for each collagen peptide were performed. Comparison of the experimental *I(Q)* curve with the theoretical curves from the models in each simulation enabled the identification of the best-fit solution structural models for each peptide. A 50 ns MD simulation at 300 K produced 12,000 models with stereochemically correct conformations for each of the nine collagen peptides (Table 4). These ensembles included a range of triple helical conformations, as well as including nonlinear molecules. Further MD simulations at 400 K did not affect the distribution of the calculated *R*_*g*_ values and indicated that the simulations had reached equilibrium.

**Figure 9:**
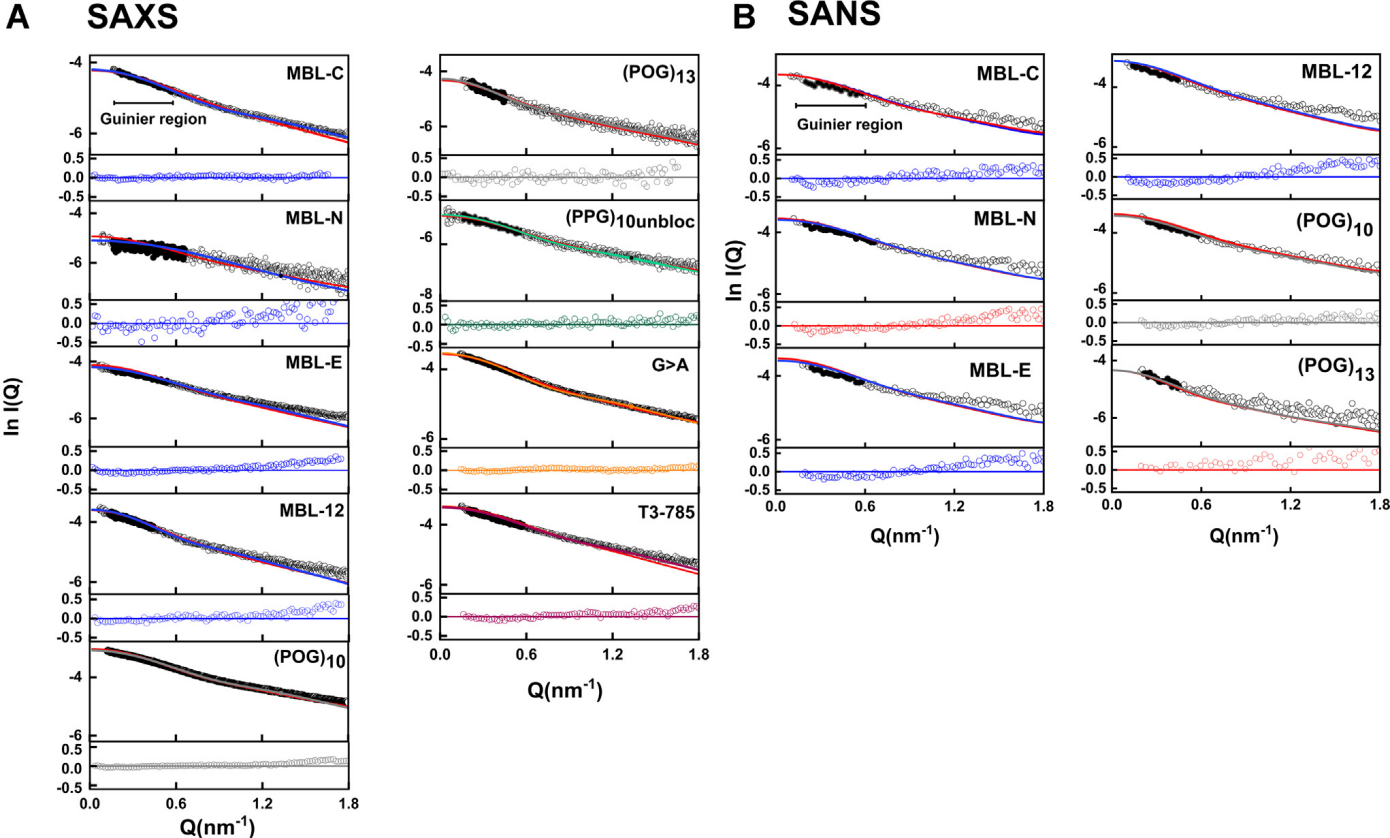
**Comparison of the crystal derived linear and best-fit MD modeled curves with the experiment.***A* and *B*, the superimpositions of the linear and best-fit MD *I(Q)* scattering curves with the experimental curves are shown for the SAXS data (*left*) and SANS data (*right*). *Black* represents the experimental data, *red* represents the linear model curves, and *blue*, *gray*, *green*, *orange,* and *pink* represent the MD-modeled curves using the color scheme of Figure 1. The *filled circles* and *horizontal bars* represent the Guinier *Rg* range. Underneath each panel, the residuals of the curve fits compared to each modeled curve are shown. MBL, mannan-binding lectin; POG, the tripeptide sequence Pro-Hyp-Gly; MD, molecular dynamics; SANS, small angle neutron scattering; SAXS, small angle X-ray scattering.

**Figure 10:**
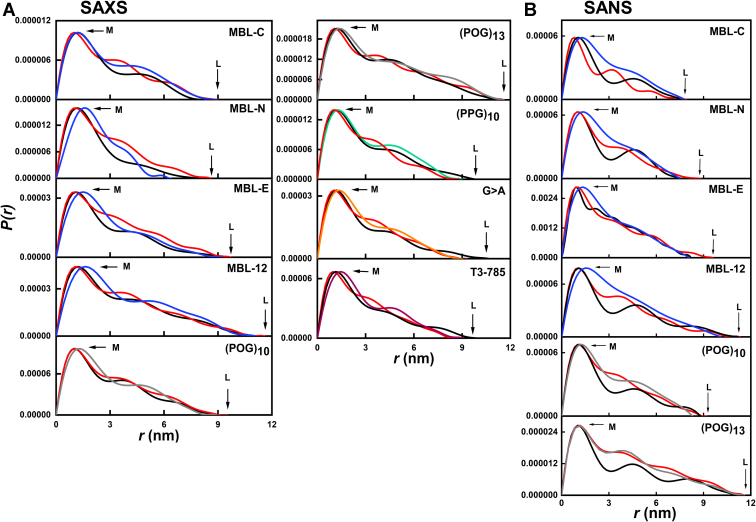
**Comparison of the crystal derived linear and best-fit MD modeled curves with the experiment**. *A* and *B*, the superimpositions of the linear and best-fit MD *P(r)* scattering curves with the experimental curves are shown for the SAXS data (*left*) and SANS data (*right*). *Black* represents the experimental data, *red* represents the linear model curves, and *blue*, *gray*, *green*, *orange,* and *pink* represents the MD-modeled curves using the color scheme of Figure 1. MBL, mannan-binding lectin; POG, the tripeptide sequence Pro-Hyp-Gly; MD, molecular dynamics; SANS, small angle neutron scattering; SAXS, small angle X-ray scattering.

The 12,000 models for each of the nine collagen peptides were converted to their hydrated and unhydrated coarse-grained sphere representations for calculation of the scattering curves, and their comparison with the experimental SAXS and SANS curves, respectively, using SCT software (35) (Figs. 8 and 9). Python-based SCT scripts were used to automate the optimization of the MD models (sphere box side and hydration cut-off) and to execute the constrained modeling analysis workflow. A theoretical *I(Q)* curve for each of the 12,000 collagen models was computed using experimental curve parameters using SCT software (35). The output also yielded comparison of each of the 12,000 modeled *I(Q)* curves with the experimental *I(Q)* curve through the goodness-of-fit *R* factors (Fig. 10). The quality of the curve fits is reported in terms of the R-factor values (Methods). As in protein crystallography, lower *R* factors represented the better fit structures. The independent SAXS and SANS fits are each considered in turn:

(i) The nine SAXS fits each resulted in a clustered distribution of *R* factors whose *R*_*g*_ values at the minima were within range of the experimental SAXS *R*_*g*_ values (denoted by *vertical dashed lines*). That for MBL-N gave higher *R* factors for reason of the noisy experimental SAXS data (Fig. 10*A*). The MD structures gave notably lower *R* factors (blue) than the linear model (red) for the four MBL peptides, showing that better curve fits were obtained from many MD structures compared to the linear starting models (Table 4). For the (POG)_10_, (POG)_13_, (PPG)_10_, G > A, and T3-785 peptides, the *R* factors for four of the five of the best-fitting MD structures were slightly lower than those for their linear starting models, indicating that the MD fits had mostly given improved structures compared to the linear starting models.
(ii) The six SANS fits again resulted in clustered distributions whose minima were within range of the experimental SANS *R*_*g*_ values (Fig. 10*B*). Three of the four MD best-fit MBL structures gave R-factors (blue) that were less than those for the four linear starting models (red) (Table 4). The (POG)_10_ and (POG)_13_ fits were likewise improved by the use of the MD curve fits.

The theoretical *I(Q)* curves from the best-fit MD-modeled structures were now compared against their experimental *I(Q)* curves (Fig. 9). Visually the MD fits showed better fits to the experimental data curves (black) than the curves from the linear models (red). The largest difference was found with MBL-N, where the MD fits gave improved fits to the experimental curves. The same outcome was found for the *P(r)* curve comparisons of the experimental data and the linear and MD models (Fig. 10). Generally, many of the three sets of curves showed similar appearances. Visually, the largest difference was found in the SAXS curves for MBL-N, when the MD fit (blue) was closer to the experimental curve (black) than the linear model (red).

**Figure 11:**
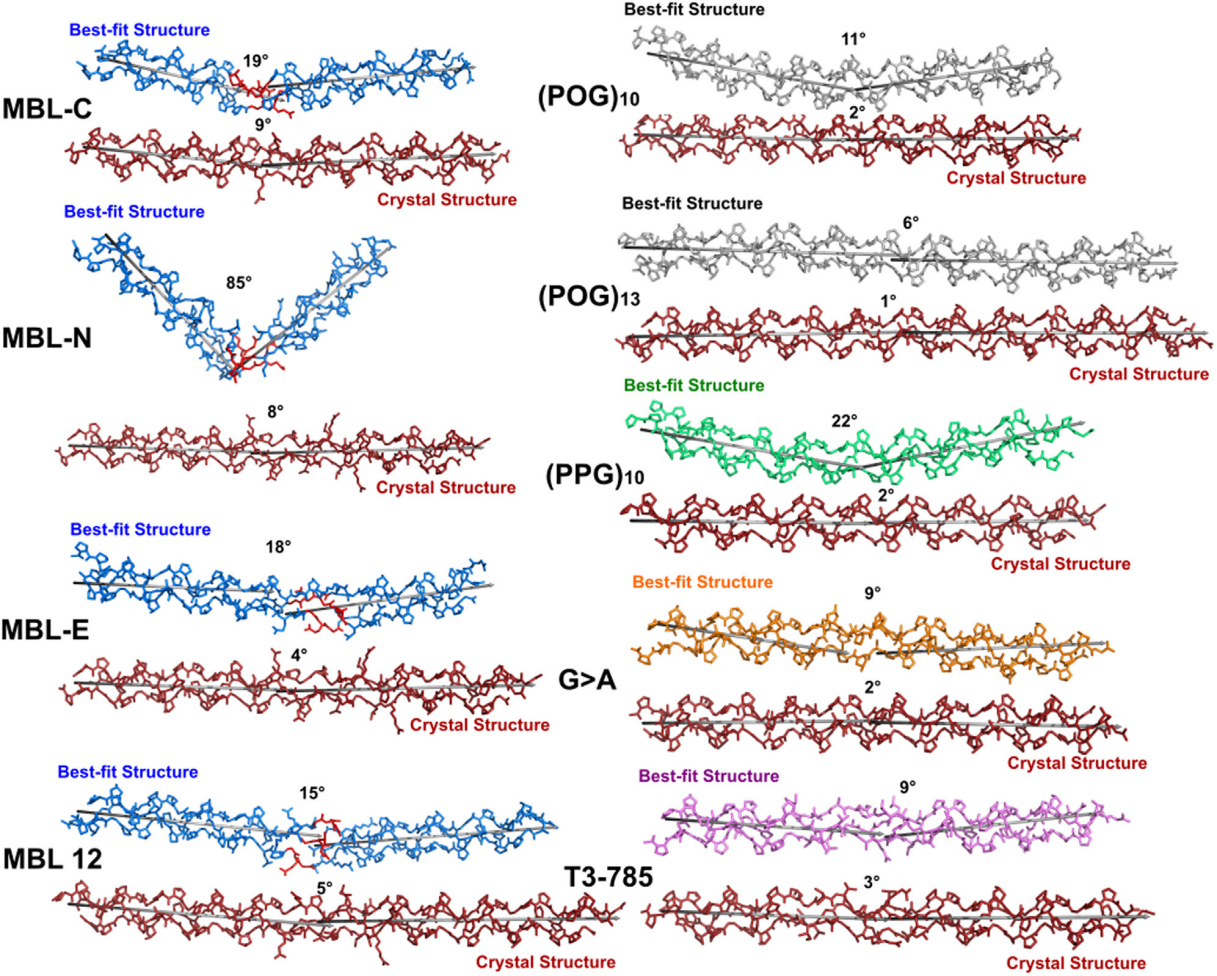
**Best-fit MD and linear crystal-derived structures of the collagen peptides.** The best-fit MD structures and the linear crystal-derived structures are shown in pairs for the MBL-C, MBL-N, MBL-E, MBL-12, (POG)_10_, (POG)_13_, (PPG)_10_, G > A, and T3-785 collagen peptides. *Blue*, *gray*, *green*, *orange,* and *pink* represent the MD-modeled curves using the color scheme of Figure 1. The GQG interruption in the four best-fit MBL peptides is highlighted in *red*. The linear models are shown in *red*. Note that the (PPG)_10_, G > A and T3-785 linear structures corresponded to their crystal structures and not to homology-modeled structures as for the MBL peptides. The angles were determined using the anglebetweenhelices.py module in PyMol. MBL, mannan-binding lectin; POG, the tripeptide sequence Pro-Hyp-Gly; MD, molecular dynamics.

**Figure 12:**
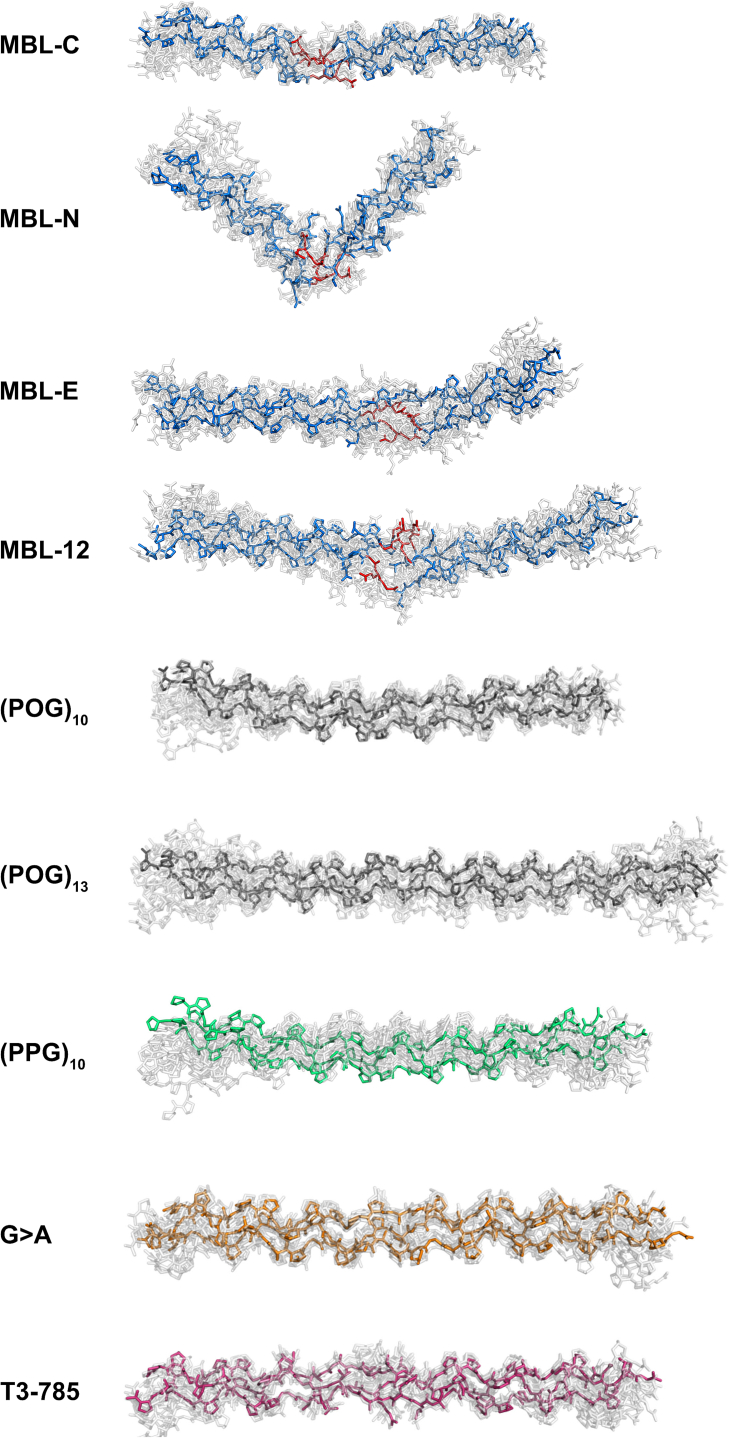
**Overlap of the top ten MD models for the collagen peptides that were best-fitted to the experimental scattering curves.***Blue*, *gray*, *green*, *orange*, and *pink* represent the MD-modeled curves using the color scheme of Figure 1, while the remaining nine are *grayed*. The GQG interruption in the four MBL peptides is highlighted in *red*. MBL, mannan-binding lectin; POG, the tripeptide sequence Pro-Hyp-Gly; MD, molecular dynamics.

Views of the nine linear and SAXS and SANS best-fit MD collagen structures clarified the outcome of the curve fits. The same best-fit structures were identified in both the SAXS and SANS fit analyses, indicating the consistency of the MD analyses. For the four MBL peptides, the MD models showed localized loosening at the GQG interruption sites in the MBL peptides as expected, where bending occurred, and the overall triple helix structures were maintained (Fig. 11). Most striking was the large bend of 85^o^ seen in both the SAXS and SANS best-fit structure for MBL-N which corresponded to the native MBL sequence with eight residues EPGQGLRG flanked by POG repeats. Smaller bends of 19°, 18°, and 11° were seen at the GQG interruptions at the centers of the other three MBL peptides MBL-C, MBL-E, and MBL-12. In contrast, the four MBL models derived from crystal structures showed smaller bends of 4°-9°. For the (POG)_10_, (POG)_13_, (PPG)_10_, G > A, and T3-785 structures, there were reduced differences between the linear starting models (bends of 1° to 3°) and the experimental best-fit structures of 6^o^-11^o^, apart from an increased bending of 22° seen for the best-fit (PPG)_10_ structure (Fig. 11). To confirm that these structures corresponded to the best fit MD structures, the 10 best-fit structures were superimposed upon each other. Each peptide consistently showed similar structures in the best fits (Fig. 12).

As a further check of the MD modeling analyses, the *s*_*20,w*_ values of the 10 best-fit MD structures were calculated using HYDROPRO for comparison with their experimental *s*_*20,w*_ values (Table 2). Overall, the mean *s*_*20,w*_ values of the linear structures were lower than the experimental *s*_*20,w*_ in light water (Table 2). This indicated that the experimental data were consistent with less elongated structures than the linear structures used in calculations. The *s*_*20,w*_ values calculated from the best-fit MBL models were larger than those from the linear models and closer to the experimental values to within ±0.21 S (Experimental procedures) than those calculated from the linear structures. This outcome confirmed that the four MBL peptides were bent, in distinction to most of the other six collagen peptides and their linear starting models.

## Discussion

Structural flexibility or bending in some types of collagen and in the collagens of the immune system proteins has been reported and appears central to their functional role, as discussed previously (19, 36, 37). The combination of AUC, SAXS, SANS, and MD simulations have uncovered new molecular details of the MBL collagen region. This approach was applied to collagen peptides that represented the GQG interruption in the triple-helix region of MBL. To benchmark this work, this approach was also applied to model (POG)_n_ peptides, as well as to three collagen-like peptides with distinctive destabilizing features and published high-resolution crystal structures. Interestingly, the scattering data for several of the MBL peptides fitted well with clearly bent structures, while the best-fit atomistic models for the non-MBL peptides were generally close to their linear crystal structures.

Small sequence changes can have a large effect on the best-fit solution structures. The POG triplets are very stabilizing and are relatively rigid due to the two imino acids. It is not possible to predict how the region around the GQG interruption in the MBL peptides will behave if additional POG rigid triplets are included. Our observations indicated that the bending is less when additional POG triplets are added to both ends in MBL-12, suggesting that these extra triplets make it energetically less favorable for a large bend to occur. In relation to the difference between MBL-N and MBL-E, where there is a large change in the shape, it is interesting to note that the Pro in the Y position of collagen XYG triplets is almost always hydroxylated, but, surprisingly, amino acid sequencing showed that the Pro preceding the interruption GE**P**GQG was not hydroxylated in the MBL protein. Hyp is known to provide more triple-helix stability than Pro, so the observation that MBL-N has a lower thermal stability than MBL-E is expected. But the observation that the presence of Hyp seems to make the formation of the sharp kink less favorable is a novel observation and may suggest that the stabilizing effect of Hyp on the triple-helix makes the helix less likely to bend.

The combined experimental and atomistic modeling approach was applied successfully to all the triple-helical peptides in this study. Thermostability experiments confirmed that all the peptides formed stable triple helices. Interestingly, the stability of the (POG)_10_ peptide was much reduced from a T_m_ value of 64 °C to one of 32 °C by replacing a central POG triplet by the GQG interruption in MBL-C, showing the destabilization caused by the GQG interruption. Replacing three POG triplets with the MBL sequence EPGQGLRG in MBL-N further lowered the T_m_ from 32 °C to 21 °C. Increasing the number of POG triplets as in MBL-11 stabilized the MBL-N structure. This study thus examined whether this loss of stability is reflected in changes in the nonlinearity of the triple helix. The structural monodispersity and size distribution of the 10 collagen peptides was established from AUC. The experimental SAXS and SANS data analyses provided their hydrated and unhydrated overall dimensions. A small family of best-fit structures was filtered from comparing 12,000 physically realistic MD models of the triple helix peptides against the experimental SAXS and SANS data. This approach follows that previously employed for antibodies, other proteins, and a series of (POG)_n_ peptides (19, 20, 38). Despite the low masses of the peptides, which ranged between 8.0 and 11.5 kDa and which do not favor good signal-noise ratios in solution scattering, the scattering data could be well fitted with clearly bent MD structures for the four MBL peptides. MBL-N with the lowest proportion of (POG) triplets and the lowest T_m_ value revealed a pronounced V-shaped conformation (Fig. 11).

The remaining collagen peptides provided benchmarks for the MBL peptide studies. Compared to the four MBL peptides, the best-fit models for (POG)_10_ and (POG)_13_ were comparatively linear with bends of 6° and 11° and show the role of the imino acids in structural stability. The synthetic collagen peptide (PPG)_10_ with no hydroxyproline residues displays reduced thermal stability (melting temperature T_m_ 24.5 °C) compared to (POG)_10_ (T_m_ 60 °C). The thermal stability of (PPG)_10_ is thus dramatically lower than (POG)_10_, consistent with the stabilizing stereoelectronic and enthalpic effects of Hyp (14), and the larger bend of 22° seen for (PPG)_10_ compared to (POG)_10_ and (POG)_13_. Interestingly, the high resolution crystal structure of the (PPG)_10_ triple helix is indistinguishable from that for (POG)_10,_ both having a bend of 2° (26, 28, 29), while the solution data show that the (PPG)_10_ and (POG)_10_ peptides differ significantly in their linearity (19).

The best-fit atomistic solution models for the G > A peptide was close to its linear crystal structure. Missense mutations which replace one Gly by a larger residue lead to a range of hereditary disorders. For instance, Gly substitution mutations in type I collagen are the most common cause of osteogenesis imperfecta, a hereditary disorder characterized by bone fragility (39). The Gly->Ala peptide models such a missense mutation in a (POG)_10_ context, and this break in the repeating tripeptide pattern results in a major loss of triple-helix stability (28). The crystal structure of G > A shows a linear triple-helical structure with local unwinding at the Gly to Ala substitution site, where several direct interchain hydrogen bonds are replaced by water mediated hydrogen bonds (9). The hydrodynamic/modeling studies produced a best fit model in good agreement with the linearity seen in the crystal structure, suggesting that the replacement of a Gly by Ala in this context did not affect the linearity of the triple helix in solution.

The T3-785 peptide, with a nine-residue imino acid-poor sequence derived from Type III collagen, gave a near-linear structure with loose imino acid-poor regions in line with the crystallographic studies (25, 40). This indicates that a locally destabilizing sequence in collagen does not necessarily translate into bending, even near the unique collagenase cleavage site.

In distinction to sequence variations in the triple helix, the molecular modeling of the standard (POG)_10_, (POG)_13_, and (POG)_14_ peptides resulted in slightly bent structures under near physiological conditions in contrast with the starting linear crystal structures. These structures showed similar bending compared to our previous solution structures for (POG)_6_, (POG)_8_, (POG)_10_, and (POG)_12_ (10). The hydrophobic interactions of stacked prolines in triple helix backbone chains are assumed to confer stability while the side chains of Pro and Hyp residues are exposed to solvent (41, 42).

Functionally, sequence variability in the collagens can affect recognition and/or conformation in disease-causing mutations of the complement proteins. The MD approach (Fig. 10) offers a means of characterizing these sites in molecular terms and testing these against experimental structural data to validate them. For the MBL collagens, the current experimental results showed that more structural nonlinearity was observed in solution than in our earlier study of the POG repeats (19). The GQG interruption together with the GEP triplet N terminal to it was associated with triple-helix destabilization and dramatic molecular bending. Bends and kinks of the magnitude reported here for MBL-N have been reported in electron microscopic studies of type IV collagen, at the MMP cleavage site in type I collagen (43), in C1q (44), and for some osteogenesis imperfecta collagens (9). Together with our C1q study (45), where an extra amino acid in the C1q collagen stalks caused a kink that was seen in solution by scattering modeling, this is the first time that such a bend in the triple-helix caused by a collagen interruption has been confirmed in solution by atomistic modeling. Previously, a collagen model for MBL was proposed with linear stalk-like collagen stems (46). This proposed MBL structure will need to be revised, and the bending at the GQG interruption will have implications for how the MASP proteins interact with MBL to activate the complement lectin pathway. However, the present experimental data show that the introduction of a GQG break in the collagen sequence is enough to induce bending in solution. This was visually clearer for MBL-N (bend 85°) than for MBL-C and MBL-E and MBL-12 (bends of 19°, 18° and 11° in that order). Interestingly, the replacement of the native Pro residue by Hyp prior to the GQG sequence in MBL resulted in less bend in MBL-E. The failure of prolyl-4-hydroxylase to recognize and hydroxylate the Pro in the Yaa position of the GEP triplet in native MBL is likely due to the adjacent GQG sequence. This retention of the unmodified Pro residue results in the dramatic kinking of MBL-N. Thus, the GEPGQG sequence directs the kink formation that is likely to play an important role in MASP binding (Fig. 11).

## Experimental procedures

### Peptide synthesis of the collagen peptides

Ten highly purified (>98%), monodisperse collagen peptides (MBL-C, MBL-N, MBL-E, MBL-12, (POG)_10_, (POG)_13_, (POG)_14_, (PPG)_10_, G > A, and T3-785), which were unblocked or blocked at both termini (acetylated N terminus and amidated C terminus), were synthesized using FastMoc Chemistry at the Tufts University Core Facility. Peptides were purified by HPLC, and their identity was confirmed by matrix-assisted laser desorption/ionization-time of flight mass spectrometry. Peptide concentrations were determined by an ultraviolet-visible spectrophotometer (Aviv Biomedical) with an extinction coefficient ε_Tyrosine 280_ of 1280 M^−1^ cm^−1^. Other peptides were purchased from Pepceuticals Ltd and Peptide Institute Inc. All peptides were obtained as a dry powder and used without any further purification (Fig. 1 and Table 1). When required, peptide concentrations were also measured by absorbance at 214 nm using ϵ_214_ = 2200 cm^−1^ M^−1^ per peptide bond.

### CD studies of the collagen peptides

CD spectra of the peptides were acquired on an Aviv Model 430 CD spectrometer (Aviv Biomedical). Wavelength scans were collected from 190 to 260 nm at 0 °C. Temperature scans were monitored by measuring the molar residue ellipticity at 220 nm from 0 to 70 °C with a 10-s averaging time and 1.5-nm bandwidth. Samples were equilibrated for 2 min at each temperature, and the temperature was increased at an average rate of 0.1 °C/min. The melting temperature (T_m_) is defined as the temperature at which the fraction-folded F(T_m_) is equal to 0.5 as described previously (47). Melting curves were obtained by measuring the CD signal at 226 nm from 0 to 70 °C with an average heating rate of 0.1 °C min^–1^ (3). As described previously, the thermal transitions at this slow rate approximate equilibrium but do not fully reach it. The T_m_ values obtained under these conditions reflect well the comparative thermal stability of different triple-helical peptides (26).

### Differential calorimetry study of the collagen peptides

DSC profiles of recombinant collagens were obtained on a NANO DSC II model 6100 (Calorimetry Sciences Corp). Each sample was dialyzed against PBS overnight before measurement. Dialysis buffer was collected and used as reference for the corresponding sample. Samples were loaded into the cells at 0 °C and heated at a rate of 1 °C/mm till 70 °C.

### Sedimentation velocity data and analysis of the collagen peptides

Sedimentation velocity AUC experiments were performed using two Beckman XL-I analytical ultracentrifuges (Beckman Coulter Inc) equipped with an eight-hole AnTi50 rotor with standard double-sector cells with column heights of 12 mm. Prior to experiments, the peptides were allowed to assemble into triple helices by allowing them to equilibrate in solution for at least 48 h at room temperature. The samples were placed in velocity cells with two-sector aluminum centerpieces. Sedimentation was monitored at a rotor speed of 50,000 rpm for ∼14 h using absorbance and interference optics at 222 nm.

The AUC data were collected at 20 °C in either 137 mM PBS prepared in deionized H_2_O (137 mM NaCl, 2.7 mM KCl, 1.4 mM KH_2_PO_4_, 4.3 mM NaH_2_PO_4_, pH 7.4) in H_2_O, 20 mM L-histidine (20 mM L-histidine, 138 mM NaCl, 2.7 mM KCL pH 6.0) in H_2_O, or in 20 mM L-histidine buffer with ^2^H_2_O. The samples were dialyzed using dialysis cassettes (2000 MWCO, Thermo Scientific Slide-A-Lyzer) with three buffer changes. With H_2_O, the concentrations of 10 peptides used in AUC experiments ranged between 0.25 and 1 mg/ml for MBL-C, 0.25 and 1 mg/ml for MBL-N, 0.25 and 1.25 mg/ml for MBL-E, 0.25 and 1.25 mg/ml for MBL-12, 0.25 and 1 mg/ml for (POG)_10_, 0.20 and 0.75 mg/ml for (POG)_13_, 0.40 and 0.80 mg/ml for (POG)_14_, 1 and 2.5 mg/ml for (PPG)_10_, 0.40 and 0.95 mg/ml for G > A, and 0.3 and 1.1 mg/ml for T3-785, respectively. The concentrations of six peptides used in the AUC experiments in ^2^H_2_O ranged between 0.94 and 3.7 mg/ml for MBL-C, 1.5 and 4 mg/ml for MBL-N, 0.75 and 4 mg/ml for MBL-E, 1 and 4 mg/ml for MBL-12, 1 and 4 mg/ml (POG)_10_, and 1 and 3.5 mg/ml for (POG)_13_. Buffer densities were measured using an Anton Paar DMA 5000 density meter. At 20 °C, the densities were 1.00543 g/ml and 1.00578 g/mol for the 137 mM PBS and 20 mM L-histidine buffers, respectively. The viscosities of the buffers were measured using an Anton Paar AMVn automated microviscometer. The viscosities at 20 °C were 0.01683 P and 0.01019 P for 137 mM PBS and 20 mM L-histidine buffers, respectively. The density and viscosity for 20 mM L-histidine buffer in ^2^H_2_O at 20 °C were 1.11100 g/ml and 0.01384 P, respectively.

The size distribution *c(s)* analyses were performed using SEDFIT software version 15.10 b (48, 49) to determine sedimentation coefficients from the interference data. Up to 35 sedimentation boundary scans (every third scan) were fitted directly to the Lamm equation in order to obtain the *c(s)* analyses which provided the sedimentation coefficients *s*_*20,w*_ and mass values. The *c(s)* analyses fitted the sedimentation data using the Lamm equation, which defines the transit of a solute under a gravitational force in a sector shaped cell. Prior to analysis, the partial specific volumes $\bar{v}$ of the collagen peptides were not known; therefore, the partial specific volumes were calculated with the program SLUV (50). However, SLUV does not support partial specific volume estimates for proteins with non-native amino acids, and the parameters for proline (Pro) were used for hydroxyproline (Hyp). The calculated $\bar{v}$ values were 0.730 ml/g for MBL-C, 0.729 ml/g for MBL-N, 0.730 ml/g for MBL-E, 0.731 ml/g for MBL-12, 0.735 ml/g for (POG)_n_ and (PPG)_10_, 0.738 ml/g for G > A, and 0.741 ml/g for T3-785 collagen molecules. The *c(s)* algorithm assumes that all species have the same frictional ratio *f/f*_0_ within each fit. To optimize the fits, the values for *f/f*_*0*_, (from an initial value of 1.2), the meniscus, the bottom of the cell and the base line were floated, until the overall root mean square deviation of the *c(s)* fits were below 0.01, and the visual appearance of the fits were reasonably satisfactory at low resolution (set at 50) and confidence (F) ratio (0.68). The final SEDFIT analyses used a fixed resolution of 200 and F ratio of 0.95. The observed *s* values were corrected to *s*_*20,w*_ by:

$$s_{20,w}=s_{T,B}\left(\frac{\eta_{T,B}}{\eta_{20,w}}\right)\frac{{\left(1-\bar{v}\rho \right)}_{20,w}}{{\left(1-\bar{v}\rho \right)}_{T,B}}$$

where *s* is the sedimentation coefficient, the subscripts _*T,B*_ refers to the temperature of the buffer. _*20,w*_ refers to water at 20 °C. *ρ* is the solvent density, *η* is the solvent viscosity, and $\bar{v}$ is the protein partial specific volume.

### X-ray and neutron scattering data and analyses for the collagen peptides

SAXS experiments were conducted in three beam sessions on Instrument B21 at the synchrotron facility of the Diamond Light Source at the Rutherford Appleton Laboratory, operating with a ring energy of 3.0 GeV (51). Samples were manually loaded into a 96-well plate and placed into the EMBL Arinax sample holder of the BioSAXS robot (52, 53). The experimental conditions, including temperature, sample locations in the plate, and data acquisition parameters, were specified in the control system. An automated sampler injected 30 μl of sample from the plate into a temperature-controlled quartz cell capillary (10 μm thick; 1.5 mm internal diameter) positioned in front of the X-ray beam. The data acquisition started when the sample reached the X-ray beam following its injection into the capillary. The quartz capillary was enclosed in a vacuum chamber to reduce parasitic scattering from air. The total exposure time was set to 60s per sample, and the exposure time of 1s per frame yielded 60 frames for each sample. The SAXS datasets were acquired in duplicate as a control of reproducibility. The images were captured using a DECTRIS PILATUS 2M detector with a resolution of 1475 × 1679 pixels (pixel size of 172 × 172 μm) and a fixed sample-to-detector distance of 4.014 m giving a *Q* range from 0.04 nm^-1^ to 4 nm^-1^ (where *Q* = 4π sin *θ*/λ; 2*θ* = scattering angle; λ = wavelength). Potential radiation damage was monitored during data acquisition to confirm its absence. The final scattering data were collected for collagen peptides ranging from 0.25 to 8 mg/ml in 20 mM L-histidine buffer at 20 °C. After in-house data reduction, buffer measurements were averaged and subtracted, from each of the sample frame using a Java-based program SCÅTTER (version 3.0) (http://www.bioisis.net) (54). The resultant averaged and merged frames represented the SAXS curve for each sample.

SANS experiments were conducted in one beam session on Instrument Sans2d at the second target station (TS2) of the pulsed neutron source ISIS at the Rutherford Appleton Laboratory (55). A pulsed neutron beam was derived from proton beam currents of approximately 40 μA. Sans2d data were recorded using 4 m collimation, and a 4 m sample-to-detector distance that gave a *Q* range of ∼0.05 to 10.0 nm^−1^. The beam operated in time-of-flight mode with a beam diameter of 12 mm and an incident wavelength range of 0.175 to 1.65 nm. 800 μl of collagen peptide samples, each prepared at concentration between 4.0 and 5.5 mg/ml in 100% deuterated 20 mM L-histidine buffer, were measured in 2 mm-thick disc-shaped Hellma quartz banjo cells positioned in a temperature-controlled rack at 20 °C. This condition showed the collagen peptides in a high negative solute-solvent contrast (50). Data collection lasted approximately 1.0 h for the buffer and each of the six peptide samples. Each raw scattering dataset was corrected for the detector efficiencies, sample transmission, and background scattering and converted to scattering cross-section data, using the instrument-specific MANTID software (56). The MANTID data reduction steps include corrections for the *Q* resolution, *i.e.*, beam divergence effects and smearing from the shape and size of the slits, as well as the wavelength overlap in each pulse (56).

Guinier analyses of the scattering data give the radius of gyration *R*_*g*_, the molecular mass and structural dimensions. In a given solute–solvent contrast, the radius of gyration *R*_*g*_ corresponds to the average distance of each scattering elements from their center of scattering and is a measure of structural elongation. *Q* is the angular difference between the incident and scattered beams. Guinier analyses at low *Q* values (where *Q* = 4π sin θ/*λ*; 2*θ* is the scattering angle and *λ* is the wavelength) give the *R*_*g*_ value and the forward scattering at zero angle *I*(0) from the expression which is valid in a *Q.R*_*g*_ range up to 1.3 (57):

$$In\;I\left(Q\right)=In\;I\left(0\right)-{R_{g}}^{2}Q^{2}/3$$

The *R*_*g*_ values were determined using the SCT suite of open source software tools (35) and were calculated using the Guinier approximation at low *Q* values such that *Q*.*R*_*g*_ < 1.3. Since the scattering curve *I(Q)* is acquired in inverse space, the structure cannot be interpreted from visual inspection alone. Fourier transformation of the scattering intensity curve *I*(*Q*) in reciprocal space into real space gives the distance distribution function, *P(r)*. The *P(r)* curve corresponds to the distribution of distances *r* between the elements. This yields the maximum dimension of the macromolecule *L* and its most common distance vector *M* in real space.

$$P\left(r\right)=\frac{1}{{2\pi }^{2}}\int_{0}^{\infty }I\left(Q\right)Qr\;\sin\left(Qr\right)dQ$$

GNOM software (version 4.6) was used to calculate the *P(r)* curve (58).

### Generation of linear models for the collagen peptides

Starting models for the collagen triple helices were constructed from a linear crystal structure of a collagen-like peptide (PDB ID: 3B0S), which has a 7/2 helical conformation and a (GPO)_9_ repeat unit (59). First, the linear model for the (POG)_10_ trimer was created from the crystal structure by duplicating a POG triplet and joining it to (POG)_9_. Modeling was performed using PyMol software (Schrödinger, LCC). The MBL-C linear model was generated using (POG)_10_ as the starting linear model by removing a Pro residue in the fifth triplet and substituting Hyp with Gln in the fifth triplet residues in all three chains. The MBL-N linear model was created using MBL-C as the starting model by replacing the POG residues in the fifth and seventh triplets to EPG and LRG, respectively. The MBL-E linear model was created using MBL-N as the starting model by adding POG at the N terminus and substituting Pro with Hyp in the sixth triplet of all three chains. The MBL-12 linear model was created by the addition of (POG)_2_ at the C terminal of MBL-E and substituting Hyp with Pro residue in the sixth triplet of all three chains. The linear models for (POG)_13_ and (POG)_14_ were created using (POG)_10_ as the starting linear model by adding three and four POG triplets, respectively. Crystal structures for (PPG)_10_ (PDB ID: 1K6F), G > A (PDB ID: 1CAG), and T3-785 (PDB ID: 1BKV) were available (23, 24, 25) and were employed as such. N-terminal acetyl (-COOH) and C-terminal amide (-NH_2_) groups were added to all the above collagen helices to create the blocked ends except for (PPG)_10_.

### MD simulations for the collagen peptides

For MD, initial models for nine collagen helices (MBL-C, MBL-N, MBL-E, MBL-12, (POG)_10_, (POG)_13_, (PPG)_10_, G > A, and T3-785) were generated using the THeBuScr, version 1.07 software package (60). The N and C termini were then subsequently blocked with acetyl and amide groups, respectively. For the (PPG)_10_ simulation, this blocking step was omitted. The models were energy minimized using the steepest descent algorithm and then placed in a TIP3P-solvated, cubic box with an edge length 0.7 nm larger than the largest axis of the model. Following solvation, the systems were energy minimized. Then, the systems were equilibrated by running short, subsequent 1 ns simulations at 50 K, 100 K, 150 K, 250 K, and 300 K, such that the final structure from the previous simulation was used as the starting structure for the next simulation. Following temperature equilibration, a production MD was run at 300 K utilizing a 2 fs time step for a simulation time of 50 ns for each of the collagen models in order to create 12,000 models for scattering fits. Extending the simulations to longer time scales (up to 200 ns) did not qualitatively change the results of the *R*_*g*_ analyses. All MD simulations were carried out using the GROMACS v4.5.5 software package with the AMBER99sb-ILDNP force-field (61). Other simulation details were the same as described by us previously (62). The bend angles in the starting and best-fit collagen models (Fig. 11) were computed using the *anglebetweenhelices.py* module in PyMol.

### Scattering curve calculation for the collagen peptides

Scattering curves were calculated for each of the 12,000 MD snapshot structures for the nine peptides using SCT software (35). The atomic coordinates for each structure were coarse grained into sphere models, using a grid with a box side of 0.53 nm and a cut-off of four atoms. The hydration shell bound at the protein surface contributes to the SAXS curves at a similar level to the protein, and this was modeled by the addition of hydration spheres corresponding to 0.3 g of water per gram of protein (33, 46). Scattering curves were calculated using the Debye equation adapted to spheres. For the crystal-derived linear structures, the theoretical scattering curves were calculated from a coarse-grained sphere model using the Xtal2SAS module of SASSIE-web (http://sassie-web.chem.utk.edu/sassie2) and SCT software (35). To monitor the agreement between the theoretical and experimental curves, the curves were compared through the calculation of *R* factors:

$$R\;\mathrm{factor}=\frac{\sum \|∥I_{Expt}\left(q\right)∥-\eta ∥I_{Theor}\left(q\right)∥\|}{\sum \|I_{Expt}\left(q\right)\|}\times 100$$

The R-factors were used to denote the quality of the fit between two scattering curves.

Theoretical sedimentation coefficients *s*^*0*^_*20,w*_ were calculated from the atomistic models using HYDROPRO (Table 2). HYDROPRO version 10 was used to calculate the sedimentation coefficients based on the atomistic structures (33), using an atomic level shell calculation and a hydrodynamic radius of 0.29 nm of each element in the model. The Hyp residues where present were converted to Pro residues by the deletion of the O atoms, Hyp not being recognized by HYDROPRO. The AUC experimental data were compared with these calculated *s*^*0*^_*20,w*_ values to assess their divergence (Fig. 2 and Table 1). The experimental *s*_*20,w*_ values were consistently larger for the five collagen helices compared to the theoretical values. The increasing divergence between the experimental and theoretical values of up to 0.27 S with peptide length suggested that the longer helices were less extended and linear than expected and corresponded to bent structures. Because the previously reported magnitude of the differences between HYDROPRO predictions and experimental values for macromolecules of well characterized *s*_*20,w*_ values was typically ± 0.21 S (63), the low differences in the pairs of *s*_*20,w*_ values for the (POG)_n_ peptides meant that additional experiments using SAXS were needed to confirm these solution structures.

## Data availability

All data are contained within this manuscript. The 10 best-fit models for the nine peptides corresponding to the X-ray fit searches at 1.00 mg/ml (Fig. 12) are available in Supporting Information. The single best-fit collagen models were also deposited in the SASBDB database (https://www.sasbdb.org/) with the reference codes MBL-C, SASDRS2; MBL-N, SASDRT2; MBL-E, SASDRU2; MBL-12, SASDRV2; (POG)_10_, SASDRW2; (POG)_13_, SASDRX2; (PPG)_10_, SASDRY2; G>A, SASDRZ2; T3-785, SASDR23.

## Supporting information

This article contains supporting information.

## Conflict of interest

The authors declare that they have no conflicts of interest with the contents of this article.

## Supporting information

Supplemental data 1

top10-GA

top10-mbl12

top10-mblC

top10-mblE

top10-mblN

top10-pog10

top10-pog13

top10-ppg10

top10-t3785
